# Spatio-temporal transcriptome dynamics coordinate rapid transition of core crop functions in ‘lactating’ pigeon

**DOI:** 10.1371/journal.pgen.1010746

**Published:** 2023-06-08

**Authors:** Yujie Wang, Xun Wang, Yi Luo, Jiaman Zhang, Yu Lin, Jie Wu, Bo Zeng, Lei Liu, Peiqi Yan, Jiyuan Liang, Hongrui Guo, Long Jin, Qianzi Tang, Keren Long, Mingzhou Li

**Affiliations:** 1 Livestock and Poultry Multi-omics Key Laboratory of Ministry of Agriculture and Rural Affairs, College of Animal Science and Technology, Sichuan Agricultural University, Chengdu, China; 2 Animal Breeding and Genetics Key Laboratory of Sichuan Province, Institute of Animal Genetics and Breeding, Sichuan Agricultural University, Chengdu, China; 3 College of Veterinary Medicine, Sichuan Agricultural University, Chengdu, China; HudsonAlpha Institute for Biotechnology, UNITED STATES

## Abstract

Pigeons (*Columba livia*) are among a select few avian species that have developed a specialized reproductive mode wherein the parents produce a ‘milk’ in their crop to feed newborn squabs. Nonetheless, the transcriptomic dynamics and role in the rapid transition of core crop functions during ‘lactation’ remain largely unexplored. Here, we generated a *de novo* pigeon genome assembly to construct a high resolution spatio-temporal transcriptomic landscape of the crop epithelium across the entire breeding stage. This multi-omics analysis identified a set of ‘lactation’-related genes involved in lipid and protein metabolism, which contribute to the rapid functional transitions in the crop. Analysis of *in situ* high-throughput chromatin conformation capture (Hi-C) sequencing revealed extensive reorganization of promoter-enhancer interactions linked to the dynamic expression of these ‘lactation’-related genes between stages. Moreover, their expression is spatially localized in specific epithelial layers, and can be correlated with phenotypic changes in the crop. These results illustrate the preferential *de novo* synthesis of ‘milk’ lipids and proteins in the crop, and provides candidate enhancer loci for further investigation of the regulatory elements controlling pigeon ‘lactation’.

## Author summary

The provision of food by parents is essential for offspring survival in some species. Unlike most birds, pigeons produce a ‘milk’ in their crops to feed hatched squabs. Here, using a multi-omics strategy, we conducted an integrated behavioral, genomic, transcriptomic, and chromatin interactome analysis of ‘milk’ production in the crop during the pigeon breeding stages. We generated a chromosome-level pigeon genome and annotated 18,660 high confidence protein-coding genes. Examination of spatio-temporal transcriptomic profiles revealed that lipid and protein synthesis-related genes are expressed at high levels in ‘lactating’ crops, suggesting that lipids and proteins used for ‘milk’ are preferentially synthesized *de novo* in the crop. We also identified the *cis*-regulatory elements of these ‘lactation’-related genes and found that their expression is modulated by variation in promoter-enhancer chromatin-level interactions. Our data indicate that lipid- and protein-loaded crop epithelial cells proliferate rapidly and slough off during the ‘lactation’ stage to form pigeon ‘milk’. This study provides an informative reference for further investigation of the transcriptional regulation underlying pigeon ‘lactation’.

## Introduction

Pigeons (*Columba livia*) have the ability to produce a nutritive substance resembling cheese-curd, or so-called pigeon ‘milk’, in their crops to feed their young [1, 2], which is thus functionally and behaviorally similar to lactation in mammals.

The crop is an outpouching of the pigeon esophagus, in which the mucosa primarily consists of squamous epithelial cells [3]. Normally, the epithelial cells slough off the lumenal surface during cell replenishment. Upon entering the nurturing stage during breeding, the epithelial cells in the crop rapidly proliferate and accumulate large amounts of lipids and proteins [4, 5]. Masses of these cells, which resemble a milk-like substance, are then released through holocrine secretion [1, 2]. Substantial efforts have been made to characterize ‘lactation’-related alterations in the morphology and genes expression of crop and identify the functional components in the pigeon ‘milk’ [5–10]. Nevertheless, a panoramic spatio-temporal view of the transcriptome dynamics during this unusual biological process, and its regulation by reprogramming of chromatin architecture, particularly the potential regulatory elements (*i*.*e*., enhancers) responsible for ‘lactation’-related genes underpinning the transitions in crop functions, remains largely unexplored.

Overall, we generated several, large multi-omics datasets, including metabolomics, a *de novo* genome assembly, RNA-seq, *in situ* high-throughput chromatin conformation capture (Hi-C) sequencing, ChIP-seq of H3K27ac (a canonical histone mark of enhancers) and spatial transcriptomics for integrated analysis of changes in the crop related to pigeon ‘milk’ production. We identified a group of ‘lactation’-related genes and pathways in pigeon, and provides a foundational resource that allows for future in-depth functional characterization.

## Results

### 1. Dramatic morphological changes in the crop and behavioral features of ‘lactating’ parent pigeons

**Fig 1:**
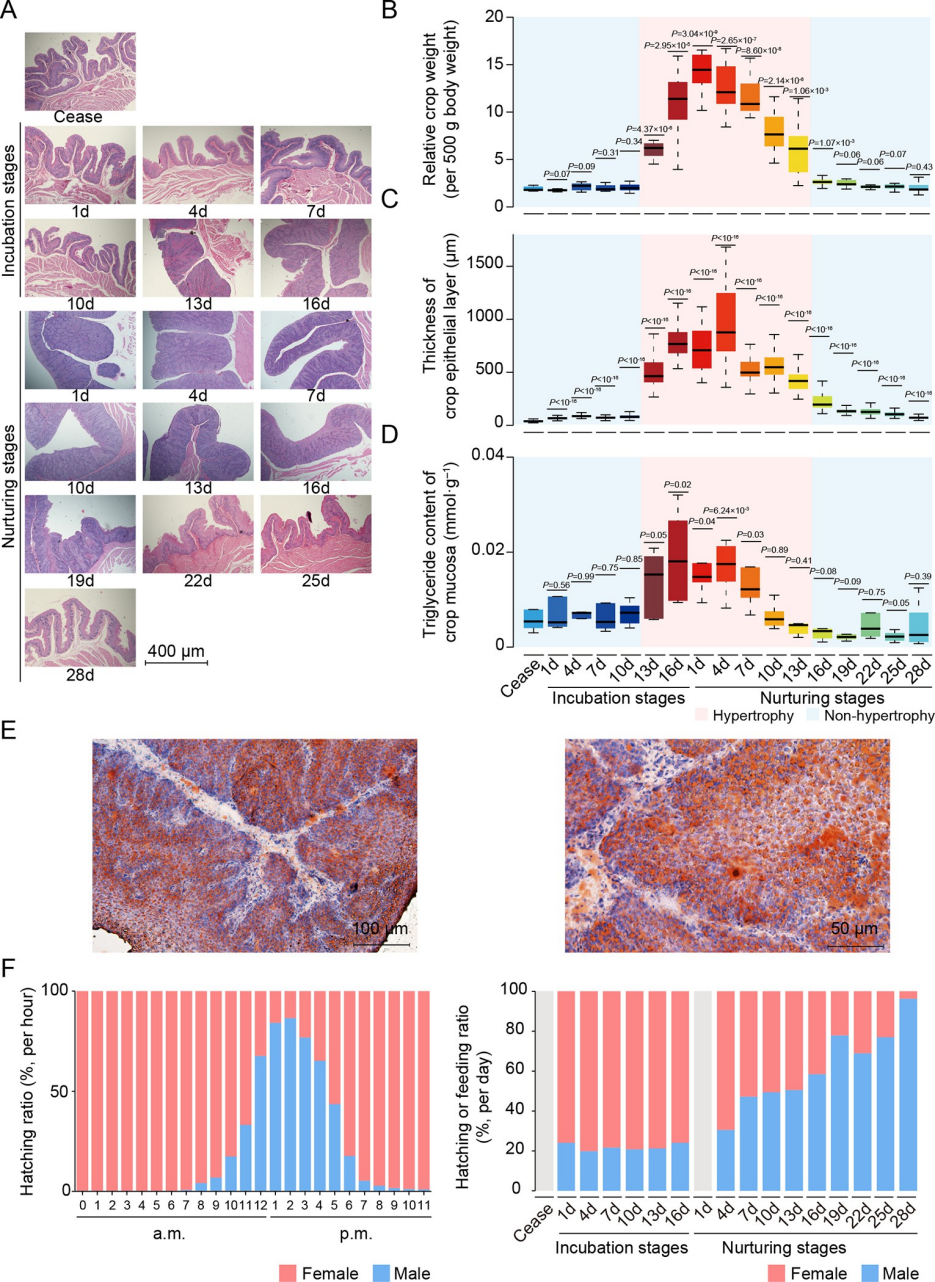
Dynamic phenotype of female pigeon crops and parental behavior during breeding stages. **A.** H & E staining of paraffin-embedded crop sections from female pigeons. **B.** Weight of the crop per 500 g body weight in female pigeons. **C.** Thickness of crop epithelium in female pigeons. **D.** Triglyceride contents of crop mucosa in female pigeons. **E.** The oil red O and counterstaining with hematoxylin of Nur1 in frozen crop sections from female pigeons. **F.** The ratio of male to female hatching per hour (left panel) or per day (right panel), and the ratio of male to female feeding per day (right panel). Stages with no available data are indicated in gray. *P*-values in **B**, **C** and **D** were calculated with Ceased stage using the Student’s *t*-test.

To characterize the histological morphology of ‘lactating’ crops, we collected crops from parent pigeons across the entire breeding stage, including the ceased stage (Ceased), six incubation stages (Incub. days 1–16) and ten nurturing stages (Nur. days 1–28) (**Fig 1A**). For female pigeons, the crop appeared as a delicate pouch at Incub1–Incub10 and Nur16–Nur28, which was reflected by its similar relative weight (RW, ratio of crop to body weight, multiplied by 500 grams, average RW: 1.76 to 2.62 g) and slightly thicker crop epithelium (average thickness: 66.30 to 194.82 μm; *P* < 10^−16^, Student’s *t*-test) than that in the Ceased stage (average RW = 1.80 g, average thickness = 37.66 μm) (**Fig 1A**, quantified in **1B** and **1C**). The primary function of the crop is food moisturizing and storage in these stages, which we designated the non-hypertrophy period of crop. By contrast, in the Incub13–Nur13 days, crops showed over 3.41-fold increase in weight (average RW: 6.14 to 14.46, *P* < 1.06×10^−3^, Student’s *t*-test) and thickened over 11.08-fold (average thickness: 417.38 to 876.24 μm; *P* < 10^−16^, Student’s *t*-test) than that in the Ceased stage. Hematoxylin and eosin staining indicated that these hypertrophy were mainly attributable to cell proliferation [6] (**Fig 1A**), and these stages were thus termed the hypertrophy period. This dramatic phenotypic change was accompanied by lipid accumulation in the crop mucosa, indicated by the higher triglyceride content in the Incub16–Nur7 days (average triglyceride content: 1.21×10^−2^ to 1.80×10^−2^ mmol·g^–1^; *P* < 0.04, Student’s *t*-test) compared with that in Ceased stage (average triglyceride content: 5.40×10^−3^ mmol·g^–1^) (**Fig 1D** and **1E**). In addition, the sloughing of cells in masses to form pigeon ‘milk’ was also observed in this stage [1, 6], and the liquid chromatography mass spectrometer (LC-MS/MS) assays indicated that lipids were enriched in pigeon ‘milk’ during this period (~68.36% of total metabolites annotated for fatty acyls, glycerolipids and phospholipids, and steroids, *etc*.) (**S1 Appendix**).

It warrants mention that male pigeons also produce pigeon ‘milk’. In this study, the crops of male pigeons showed slight and delayed changes in crop morphology compared with changes observed in females (*i*.*e*., the hypertrophy period included Incub16–Nur25 in males; average RW during hypertrophy period was 10.23 g in females and 7.05 g in males; Stages: *F* value = 104.85, *P* < 10^−16^, sexes: *F* value = 5.33, *P* = 0.02, stages×sexes: *F* value = 8.69, *P* < 10^−16^, ANOVA) (**S2-I Appendix**). Furthermore, the male hypertrophy period was accompanied by a switch in sex-specific parental behaviors in which males assumed the primary role in feeding care from females in the later stages of feeding (> 50% of the feeding time per day after Nur16) (**Fig 1F** and **S1 Video**). Whereas, the developmental characteristics of crop epithelium in histological analyses were similar between sexes during the breeding stages (**S2-I Appendix**). Moreover, the metabolites in ‘milk’ were similar between sexes (average Pearson’s *r* between sexes was 0.89, *P* < 10^−16^; only ~2.08% of annotated metabolites showed differential abundance) (**S1 Appendix**). This finding further supports the likelihood that crops followed the same developmental trajectory in male and female pigeons.

Taken together, these results indicated that the crop of parent pigeons undergoes dramatic changes in morphology during the breeding stages, in which each sex shows varying degrees of change, accompanied by a transition in parental behavior. This reflects a change in the division of labor between sexes during the breeding stages which may be related to shifts in gonadal steroid hormone levels [11, 12]. For example, in female pigeons, serum estradiol concentration increases dramatically in the later stages of feeding [13], possibly to prepare for the next breeding stages.

### 2. The transcriptional landscape of the crop across breeding stages

To establish the transcriptomic profile during crop ‘lactation’, we first generated a *de novo* chromosome-level pigeon genome, a 1.18-gigabases [Gb] genome with contigs and scaffold N50 values of 7.79 and 35.84 Mb, respectively. This represents a 290-fold increase in contig N50 size and a 10-fold increase in scaffold N50 size compared to the previous version [14] (**S3-I Appendix**). We also annotated 18,660 protein-coding genes (**S3-II Appendix**).

We then conducted comprehensive transcriptome analysis of crop mucosa across the entire breeding stage in female pigeons, resulting in 102 rRNA-depleted RNA-seq libraries, containing ~11.34 Gb of high-quality sequence data per library, or ~1.16 terabases (Tb) in total, from 17 stages with six biological replicates per stage (**S2-IIA Appendix**).

**Fig 2:**
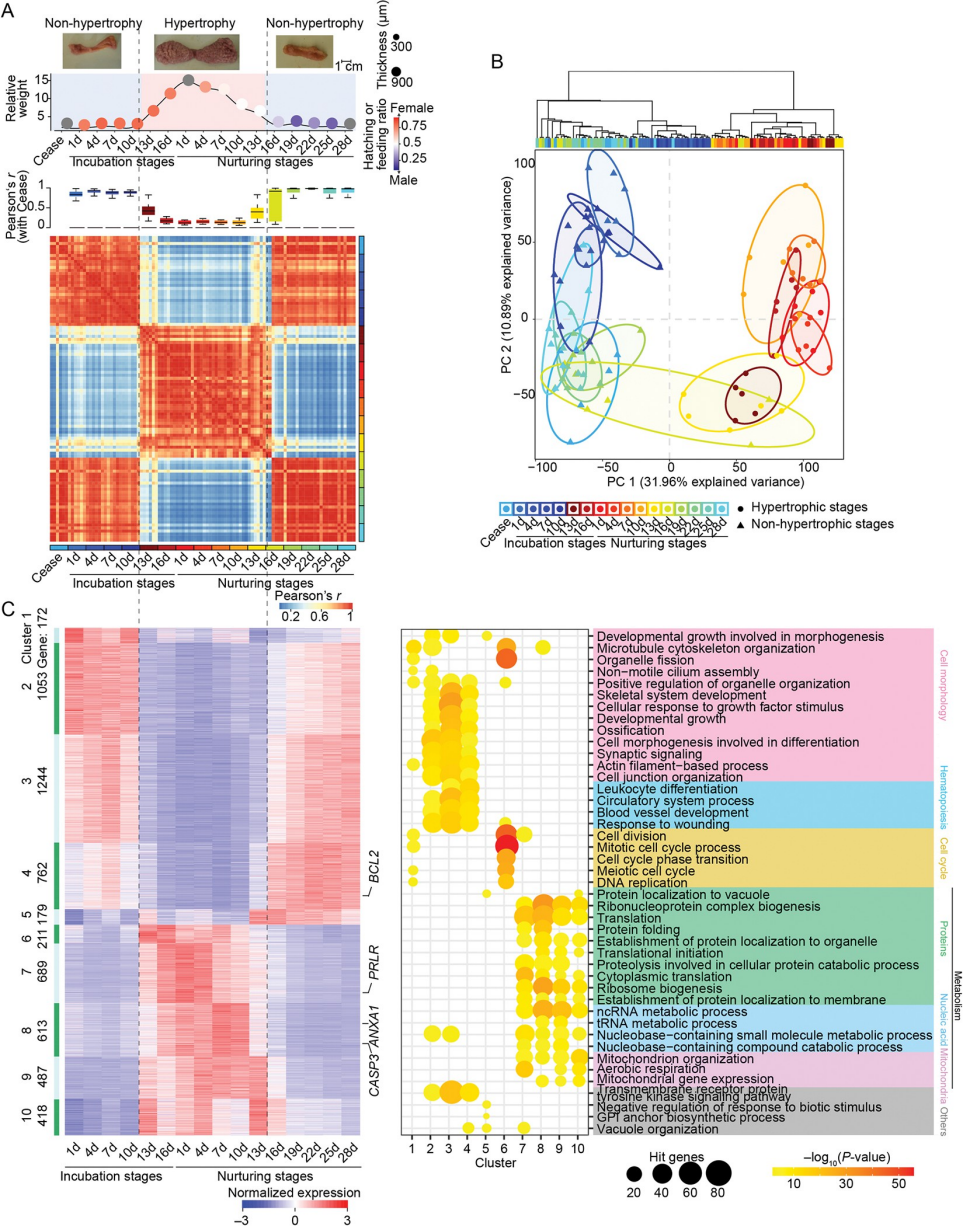
Shifts in the transcriptomic profile of the pigeon crop during breeding stages. **A.** Transcript profile remodeling is coordinated with the phenotypic changes. The phenotypic change of pigeon crops during breeding (top panel). The Pearson’s coefficients of gene expression data during breeding stages compares with Ceased stage (middle panel). The Pearson correlation coefficient matrix of gene expression profiles separated the crop samples into two pattens during breeding stages (bottom panel). Scatter plot in top panel shows crop weight (per 500 g body weight); point size indicates the thickness of the crop epithelial layer; the point color represents the ratio of male (blue) to female (red) hatching (incubation stages) or nurturing (nurturing stages) behaviors; stages with no available data indicated in gray. **B.** Unsupervised hierarchical clustering (top panel) and principal component analysis (PCA, bottom panel) of crop gene expressions during breeding stages. The line indicates the minimum volume ellipse of each biological repetition. **C.** Ten stage-specific expression clusters are revealed by *K*-means clustering (left panel). The top 5 most significantly enriched Gene Ontology-biological process (GO-BP) terms for genes in each cluster (right panel). Gene expression was normalized by *Z*-score in the heat map.

Comparison among stages revealed remarkable changes between the non-hypertrophic (*i*.*e*., Incub1–Incub10 and Nur 16–Nur28) and hypertrophic (*i*.*e*., Incub13–Nur13) periods (**Fig 2A**). Moreover, non-hypertrophic crops showed greater transcriptomic similarity with crops in the Ceased stage (average Pearson’s *r* between non-hypertrophy period and Ceased stage: 0.88, *P* < 10^−16^) than with hypertrophic crops, which showed extreme variation in gene expression compared with the Ceased stage (average Pearson’s *r* between hypertrophy period and Ceased stage: 0.25, *P* < 2.11×10^−13^) (**Fig 2A**). Hierarchical clustering and PCA plots also partitioned samples according to hypertrophy and non-hypertrophy periods (**Fig 2B**). These results suggested that the dramatic morphological changes observed in crop sections corresponded to extreme transcriptomic reprogramming (**Fig 2A** and **2B**) that reflected marked differences in female crop function among different breeding stages. It is important to note that the composition of crop cells undergoes significant change during breeding stages [15–20]. However, our bulk RNA-seq analyses only provide the average values of cell mixtures, thereby concealing cellular heterogeneity. To fully investigate the extent of cellular heterogeneity and identify which (sub-)cell types contribute to the observed differential expression in bulk RNA-seq data, it is necessary to investigate at a single-cell resolution [21].

To define dynamic patterns of gene expression that correspond to physiological, metabolic, and morphological changes in the crop throughout the breeding stages, we identified two major transcriptional programs involving 5828 genes using the maSigPro-GLM algorithm [22] (**Fig 2C**). A total of 3410 genes (Clusters 1–5) were generally down-regulated during the hypertrophy period, which enriched for categories associated with cell morphology (*e*.*g*., ‘actin filament-based process’ and ‘cell morphogenesis’) (**Figs 2C** and **S1**). Conversely, 2418 genes (primarily in Clusters 6–10) were up-regulated during the hypertrophy period, which mainly involved in processes related to metabolism (*e*.*g*., ‘protein metabolism’, ‘ribosome biogenesis’), cell cycle transition (*e*.*g*., ‘cell cycle’ and ‘cell division’) and respiration chain (*e*.*g*., ‘aerobic respiration’, ‘mitochondrial organization’), *etc*. (**Figs 2C** and **S1**). The above transcriptional profiles were corroborated at seven representative stages in male pigeons (**S2-II Appendix**), which collectively reflected a vigorous metabolism in the hypertrophic crops. Importantly, high expression of the *PRLR* (prolactin receptor) was detected in response to prolactin, a specialized protein responsible for stimulating crop ‘lactation’ in pigeons [1], in hypertrophied crops (**Fig 2C** and **S2-II Appendix, D panel**). This finding suggests the prolactin-PRLR signal pathway functions not only within the brain, for example facilitating regurgitation and feeding behavior [23, 24], but also in peripheral sites, such as the crop, to coordinately regulate cell metabolism (*e*.*g*., cell proliferation and lipid metabolism [25]) (**Fig 2C** and **S2-II Appendix, D panel**). Of note, *ANXA1*, which is specifically expressed in the prolactin-stimulated crop [26] and involved in lipid metabolism [27], was dramatically upregulated (54.19-fold, FDR = 1.03×10^−4^, calculate by edgeR based on Read abundance of gene) at Nur1 compared with its expression in the Ceased stage (**Fig 2C** and **S2-II Appendix D panel**).

Rapidly evolving genes are primary contributors to the acquisition of new functions and physiology [28–30]. Compared to genomes of the other seven ‘non-lactation’ birds, a significant proportion of the positively selected genes (PSGs, 128 of 143, or 89.51%; *P* < 10^−16^, *χ*^2^ test) and duplicated genes (2419 of 3878, or 62.38%; *P* = 9.66×10^−5^, *χ*^2^ test) in the pigeon genome have evidence of transcription in crops (TPM > 0.5 in at least one stages) (**S2A and S2B Fig**). Of them, 50 PSGs and 1672 duplicated genes exhibited alterations in expression related to temporal changes during the pigeon breeding stages (*P* < 0.05, maSigPro-GLM algorithm) (**S2B and S2C Fig**). Genes involved in the processes of ‘lipid metabolism’ (*e*.*g*., *ACAA2*, *DHCR7* and *GLIS1*) [31–34] and ‘cell division’ (*e*.*g*., *CCNE2*, *EIF3I* and *EIF3G*) [35–38] (**S2C and S2D Fig**) were consistently expressed at higher levels during the hypertrophy period compared to the Ceased stages. This is consistent with the increased nutrient (lipids and proteins) and cell renewing (replacement cycle for crop epithelium requiring only about 4 hours [5]) requirements of the crop during ‘lactation’ period. These results indicate that rapidly evolving genes in the pigeon genome may be potentially involved in shaping the ‘lactation’ of the pigeon crop.

**Fig 3:**
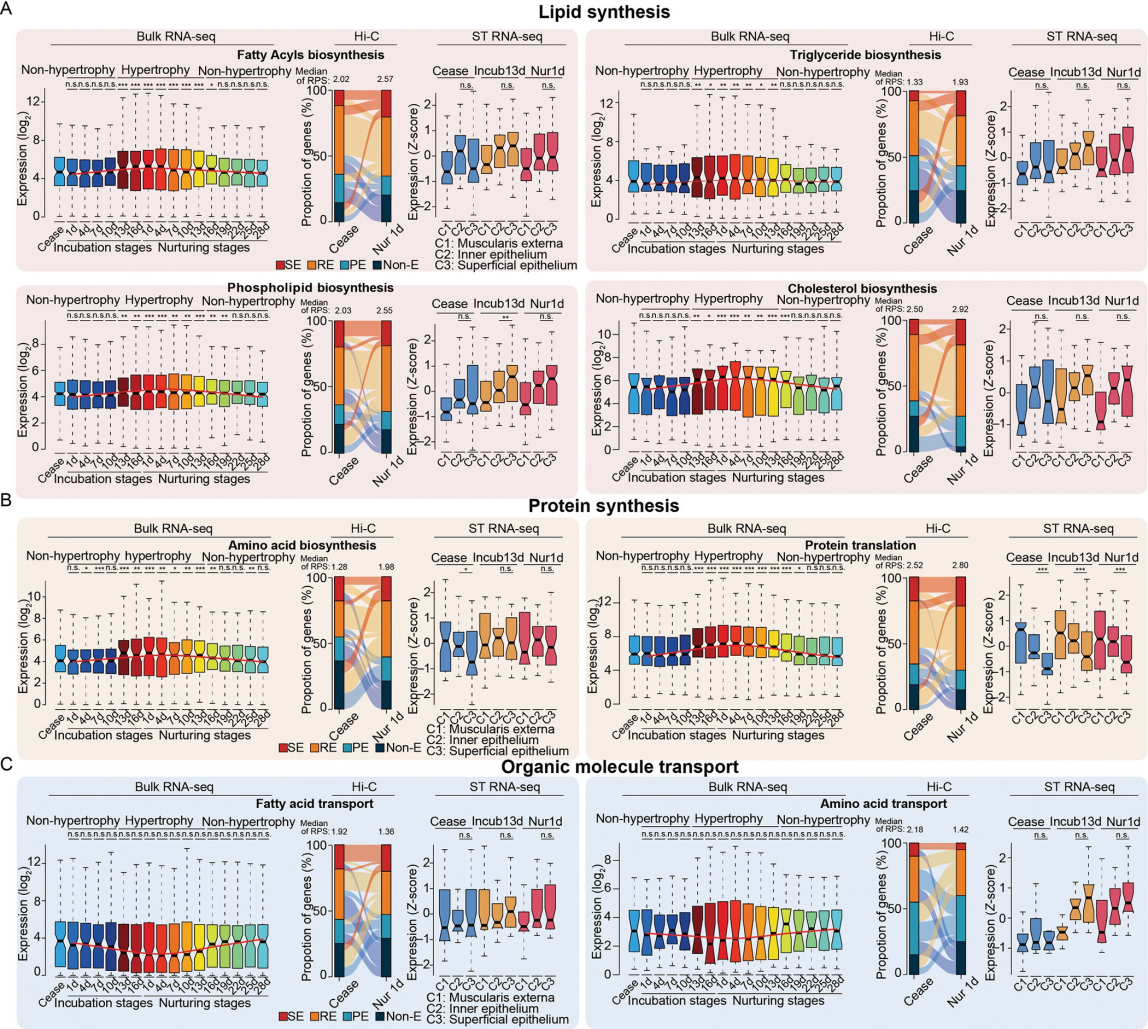
Changes in chromatin architecture and expression levels of lactation-related genes. The spatio-temporal expression profiles of genes involved in (**A**) lipid synthesis, (**B**) protein synthesis, and (**C**) organic molecule transport. Boxplots of the expression levels of each indicated ‘lactation’-related gene set in bulk RNA-seq data throughout the breeding stages are shown on the left. Alluvial plots of variation in gene contacts with different enhancer types are shown in the middle panels. Boxplots illustrating changes in the spatio-temporal distribution of expression for each indicated gene set in the spatial transcriptome data (ST RNA-seq) are shown on the right. SE, RE, PE and Non-E in alluvial plots indicate super-enhancer, regular-enhancer, poised-enhancer, and non-enhancer contacts, respectively. *P*-values were calculated for the Ceased stage (bulk RNA-seq) or between C2 and C3 (ST RNA-seq) using the Wilcoxon rank-sum test. n.s., *P* ≥ 0.05; * 0.01≤ *P* <0.05; **0.001≤ *P* < 0.01; ***P < 0.001.

**Fig 4:**
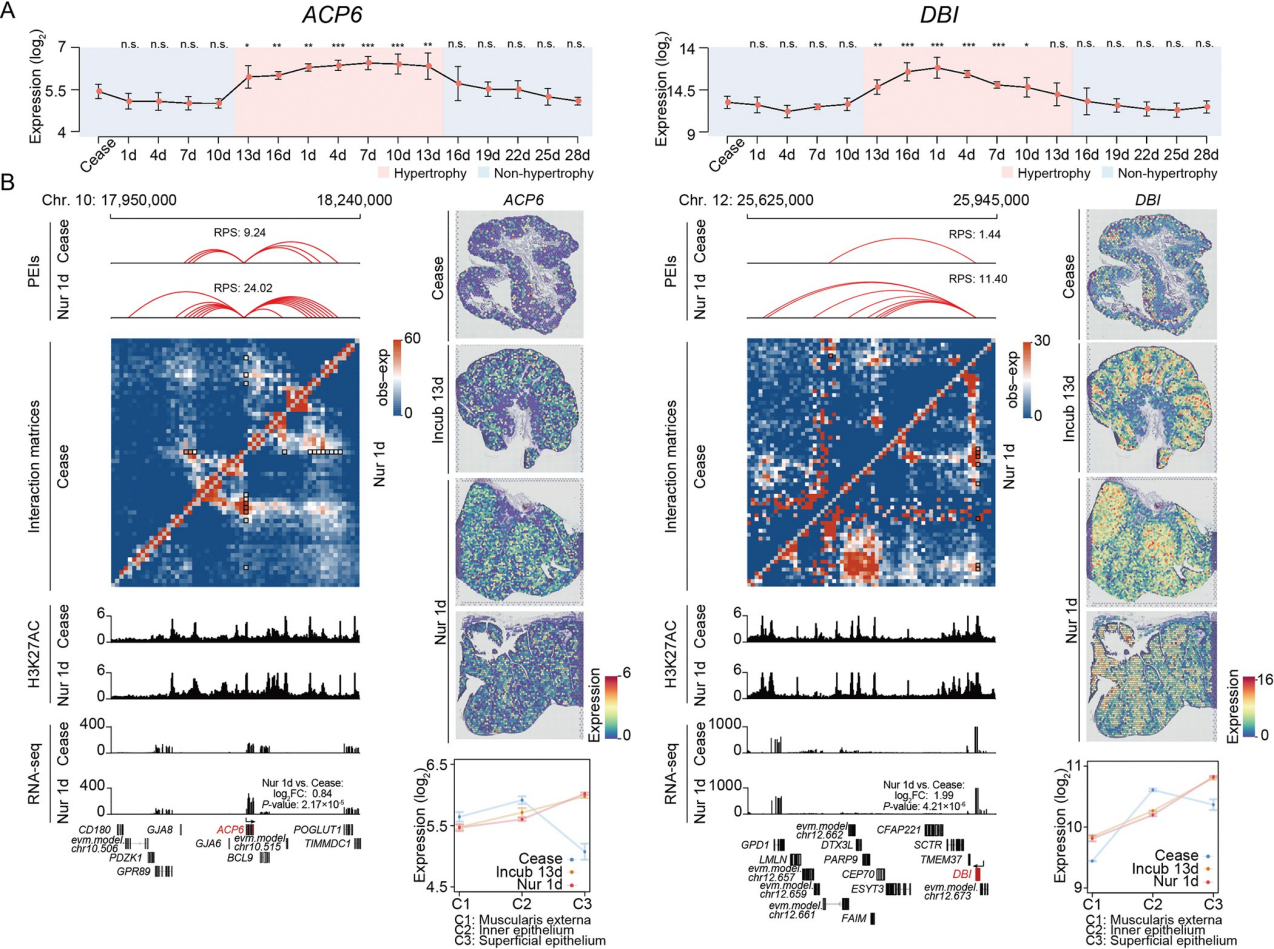
Representative crop ‘lactation’-related genes and their long-range spatial interactions. **A.** The expression levels of *ACP6* and *DBI* in different breeding stages. *P*-values were calculated by comparison for Ceased stage using Student’s *t*-test. n.s., *P* ≥ 0.05; * 0.01≤ *P* <0.05; **0.001≤ *P* < 0.01; ***P < 0.001. **B.** *ACP6* and *DBI* participate in fatty acid and phospholipid synthesis, respectively. Left: Schematics of PEIs (top), interaction matrices (upper middle), H3K27ac signals (lower middle), and transcription levels (bottom). Right: spatial distribution in three stages of breeding and expression levels in different histological layers of the pseudo bulk transcriptional profile. Log_2_FC and *P*-value in the transcription level schematic plot are calculate by edgeR based on gene abundance.

To further characterize nutrient metabolism in hypertrophied crops, we examined changes in expression for eight *a priori* representative candidate gene sets related to synthesis and transport of lipids and protein (**Fig 3**), which are the two most abundant nutrients in pigeon ‘milk’ (respectively accounting for 9–13% [lipid] and 9–11% [protein] of fresh weight [4]). As expected, genes involved in synthesis of lipid and protein were generally up-regulated in the hypertrophied crops (**Fig 3A and 3B**, left panel), such as the *de novo* lipogenesis-related gene, *ACACA* [39, 40], and lipid biosynthetic pathway genes (typically, *ACP6*, *DBI*, *GPAT3* and *SCD*) [41–45]. Consistent with the higher abundance of glutamic acid and asparagine in the ‘lactating’ crops [46], *GOT2* (involved in glutamic acid and asparagine synthesis) [47, 48] and *ASNSD1* (involved in asparagine biosynthesis) [49] were also up-regulated in hypertrophic crops (**Figs 4A** and **S3A–S1E**). In contrast, genes involved in lipid and amino acid transport generally showed a non-significant trend of down-regulation (**Fig 3C**, left panel). Nonetheless, the fatty acid transporter, *SLC27A4* [50], was significantly expressed at low levels in hypertrophied crops, while *CD36*, regulates lipid uptake by endocytosis [51–54], was significantly highly expressed (**S3F and S3G Fig**).

These results suggested a transcriptomic regulatory mechanism that could induce a rapid functional switch from food storage to nutrient production in the ‘lactating’ crop. It should be noted that the rapid proliferation of crop epithelial cells leads to a deficiency in available blood [6], which aligns with our transcriptomic data that showed markedly low expression of angiogenesis-related genes in hypertrophied crops (**S3H–S3K Fig and S2-III Appendix**), and further supported that lipids and proteins are preferentially synthesized *de novo* in the crop. In addition, we found that the highly expressed DEGs in hypertrophied crop tissue were enriched in terms related to respiration chain (**Fig 2C and S2-II Appendix**), which might reflect the accumulation of macromolecules (lipids and proteins, *etc*.) leading to the generation of reactive oxygen species [55, 56] and subsequent induction of apoptosis [57]. As expected, *CASP3* (pro-apoptotic factors) was up-regulated, while *BCL2* (anti-apoptotic factors) was down-regulated (**Fig 2C**) during the hypertrophy period. This indicate that apoptosis is known to play a key role in the sloughing of crop epithelial cells [58].

### 3. Rewiring of the spatial regulatory circuitry underpinning functional transition in the crop of ‘lactating’ pigeons

Since the chromatin architecture is an important transcription regulator, especially, the physical interactions of promoters and their long-range interacting enhancers dynamically regulate gene expression in a spatio-temporal-specific manner [59–63], we used *in situ* Hi-C to map chromatin contacts on the crop mucosa of female at two representative stages (*i*.*e*., Ceased stage and Nur1) (**S4-I Appendix**). We generated a total of ~ 576.3 million valid contacts and constructed contact maps at a maximum resolution of 2 kb by merging the contacts of the three replicates at each stage (**S4-I Appendix, A–D panels**).

The chromatin architecture was relatively stable between stages at the hierarchical structure of compartmental arrangements (7.42% of genomic regions show different compartmental status, *i*.*e*., A/B switches), partitions of topologically associating domains (TADs) (0.57% of TAD boundaries were shifted), and intra-TADs strength (8.44% of TADs exhibit changed intra-TADs interactions) (**S4-I**, **S4-II** and **S4-III Appendix**). Notably, we found 6 genes (*e*.*g*., *PRLR*) located within regions of variable intra-TAD strength that were involved in ‘regulation of STAT cascade’ (the principal pathway activated by prolactin-PRLR signaling), *etc*. (**S4-III Appendix I panel**), suggesting chromatin architectures are plastic to response crop ‘lactating’.

At a finer scale, we identified ~50,046 promoter-enhancer interactions (PEIs; median size = 115 kb in length) (**S1 Table**), which were assigned to ~8,068 promoters and primarily restricted within TADs (**S4-IV Appendix**). Next, we found that in eight *a priori* ‘lactation’-related gene sets (*i*.*e*., lipid synthesis, protein synthesis, and organic molecule transport), genes involved in lipid and protein synthesis were likely to interact with more active enhancers (measured by H3K27ac ChIP-seq signals), whereas organic molecule transport-related genes showed fewer interactions with active enhancers at Nur1 (**Fig 3**, middle panel). Combined with the observed changes in gene expression levels (**Fig 3**, left panel), these results supported that the differences in transcriptomic programs between breeding stages are due to the additive effects of the enhancer (*i*.*e*., RPS index [64]) as well as enhancer activities (**S4-IV Appendix**). Our analysis revealed that genes responsible for lipid biosynthesis (*ACACA*, *ACP6*, *DBI*, *GPAT3*, and *SCD*), amino acid biosynthesis (*ASNSD1* and *GOT2*), and lipid transport (*CD36*) had a greater number of enhancer interactions and were typically upregulated in Nur1 compared to the Ceased stage. In contrast, genes responsible for lipid transport (*SLC27A4*) with fewer enhancer interactions were generally downregulated at Nur1 compared to the Ceased stage (**Figs 4B** and **S3**).

Spatial proximity between promoters, which is established through the occupancy of a common transcription factor, constitutes an additional layer of genome organization responsible for regulating gene expression [65, 66]. Strongly expressed genes were found to exhibit an elevated degree of contacts among themselves (**S4-V Appendix**). Notably, metabolic (*i*.*e*., GFI1B [67], XBP1 [68], and CLOCK [69]) and proliferative (*i*.*e*., APC [70, 71], RB1 [72], and E2F3 [73, 74]) transcription factors were predicted to bind at genomic sites of genes that were specifically highly expressed in Nur1 (**S4-V Appendix**). Furthermore, we observed preferential interactions between the promoters occupied by STAT5, the key transcription factor in prolactin signaling [24, 75] (**S4-V Appendix**). Correspondingly, the expression levels of STAT5 target genes were slightly but significantly upregulated in Nur1 (**S4-V Appendix**). This finding provides further support for the regulatory effects of prolactin in crop ‘lactation’ [1, 2, 24].

These collective data provide evidences that long-range chromatin interactions participate in shaping the transcriptional dynamics of ‘lactation’-related genes during the breeding stages. Identification of the predicted enhancer loci (**S1 Table**) also provides an informative reference for further investigation of the regulatory elements controlling pigeon ‘lactation’.

### 4. Spatially resolved transcriptomics in the crop of ‘lactating’ pigeons

Crop mucosa consist of stratified squamous epithelial cells, including basal layer (undifferentiated cells, at the innermost of mucosa), a spinous layer (postmitotic cells), a granular layer (harboring flat cell), and a keratin layer (keratinized cells, at the surface of mucosa, which sloughs off to form pigeon ‘milk’) [3, 6, 15–20] (**S4A Fig**).

We thus used a spatial transcriptomics (ST) approach to dissect transcriptional differences in the crop, especially the crop epithelium across three representative stages: Ceased stage (*i*.*e*., non-hypertrophy), Incub13 (initial hypertrophy), and Nur1 (hypertrophy period). During the hypertrophy period, the basal layer cells grow into the suprabasal layer and multiple other cell layers at one barcoded sample site (**S4A Fig**, top panel), thus we divided the crops into muscularis externa (Cluster 1), inner epithelium (Cluster 2), and superficial epithelium (Cluster 3) layers (**S4A–S4C Fig**). These histological strata could be well resolved by UMAP spatial distribution analysis (**S4B and S4C Fig**) with layer-specific marker gene expression (including *ACTG2*, *E-FABP*, and *CSTA*, which are markers for muscle cells, spinous cells, and granular cells, respectively) [76–78] (**S4D–S4F Fig**).

We then performed “pseudo-bulking” by summing the UMI counts for each gene within each layer across each spatial replicate to build a layer-enriched expression profile (**S4G–S4I Fig**) to define the spatial transcriptomic patterns of crop ‘lactation’-related genes (**Fig 3**, right panel). In Ceased stage, genes involved in lipid and protein synthesis were preferentially expressed in the inner epithelium (**Fig 3A and 3B**, right panel), and with further progression into hypertrophy period, the expression levels of lipid synthesis genes in the superficial epithelium were comparable with the inner epithelium (**Fig 3A**, right panel). Nonetheless, protein synthesis genes, particularly translation-related genes, were also preferentially expressed in the inner epithelium (**Fig 3B**, right panel). Genes involved in lipid biosynthesis (*ACACA*, *ACP6*, *DBI*, *GPAT3*, and *SCD*) showed high expression in the superficial epithelium, whereas genes involved in amino acid synthesis (*ASNSD1* and *GOT2*) were highly expressed in the inner epithelium. However, although they were both highly expressed during the hypertrophy period (**Fig 4B** and **S3**).

These results were consistent with the accumulation of lipid droplets observed throughout the suprabasal layer (**Fig 1E**). Additionally, we observed high expression of genes related to protein translation in the inner epithelium (**Fig 3B**, right panel). This is consistent with previous research indicating that polyribosomes, which are involved in protein synthesis, were more abundant in the spinous layer [20]. This may serve as a mechanism to meet the high demand for protein translation during crop ‘lactating’.

## Discussion

The provision of food by parents is important for the growth and survival of offspring, notably exemplified by mammalian milk, a specialized food that is synthesized and secreted in an apocrine manner by epithelial cells in the mammary glands [79]. However, in some altrices (*e*.*g*., pigeons, flamingos [80], and emperor penguins [81]), fish (*e*.*g*., great white sharks [82] and discus fish [83]), or even invertebrates (*e*.*g*., jumping spiders [84]), the parents also provide specialized food for offspring.

Among non-mammals, pigeons produce a nutritive ‘milk’ in their crop which, although functionally similar, largely differs from mammalian milk in its mechanism of production, histological basis, and nutritional composition. Histologically, pigeon ‘milk’ is composed of crop epithelial cells, that proliferate rapidly (**Fig 1A**) and are holocrine to the crop lumen during hypertrophy period. However, an avian-specific cornified envelope allows holocrine secretion of these epithelial cells without complete disruption of cell morphology [85]. Nutritionally, pigeon ‘milk’ is enriched with proteins and lipids, but lacks carbohydrates [86]. Lipids accumulate during the cornification of epithelial cells [85, 87, 88], which may be the physiological basis of crop ‘lactation’. However, lipid synthesis and translocation from an exogenous source cannot be excluded, since fatty acid synthase expression in the pigeon liver also increases during ‘lactation’ [89], and subsequently, fatty acids or very-low density lipoproteins may enter crop epithelial cell via endocytosis [6]. Interestingly, the prolactin-induced, crop-specific expression of ANXA1 [26] (**Fig 2C**), a gene involved in the synthesis and transport of multivesicular bodies and endocytic vesicles [90], further supports this possibility. By contrast, the high protein content in pigeon ‘milk’ is attributable to the high expression of protein translation-related genes in the inner epithelium (**Fig 3B**), the germinal layer of the crop comprising highly proliferative epithelial cells (**S4I Fig**) [91, 92]. The holocrine mode of pigeon ‘milk’ supports the likelihood that the proteins in ‘milk’ are mainly derived from these cells.

Interestingly, ‘lactation’ in pigeons, mammals and even fishes are all regulated by prolactin [83, 93, 94], including roles in maternal behavior, immunomodulation, growth and development and metabolic regulation [25, 95]. The major targets of prolactin are epithelial cells, which recognize and respond to prolactin via prolactin receptors (PRLR) [96]. Detection of PRLR expression in the crop at different time points during ‘lactation’ (**Fig 2C**) suggested that this gene is regulated by its local genomic context, that is, variation in the extent of intra-TAD chromatin interactions (**S4-III Appendix**) and spatial rewiring of regulatory circuitry (*i*.*e*., modulating promoter-enhancer interactions) (**S1 Table**). These results thus provide a transcriptional regulatory foundation for investigating the cues and downstream regulatory signals involved in avian ‘lactation’.

In conclusion, this study highlights the dramatic morphological changes in the crop of ‘lactating’ pigeons, and illustrates how these changes are activated by remodeling the crop transcriptional profile throughout different breeding stages. Moreover, this study reveals a crucial suite of lipid and protein metabolism-related genes that are functionally associated with ‘lactation’ and regulated by modulating chromatin PEIs among lactation-related enhancers and gene loci. Our results also highlight the local histological effects of these ‘lactation’-related genes.

## Materials and methods

### Animal model construction and ethics statement

White King pigeons were used as animal models for this study. We constructed a pigeon population consisting of more than 200 males and 200 females. The pigeons were housed in individual wire-mesh cages. All experimental animals lived under the same normal conditions, with food and water given *ad libitum*. Animal maintenance and experimental procedures were approved by the Institutional Animal Care and Use Committee in the College of Animal Science and Technology, Sichuan Agricultural University, Sichuan, China under permit No. DKY-2019102015. Throughout the procedure, particular care was taken to avoid animal suffering and to ensure ethical treatment.

### Phenotypic measurements

To observe the morphological changes occurring in the crop during ‘lactation’, 4% paraformaldehyde was used to fix the crop tissue. After dehydration, paraffin embedding and sectioning, the sections were stained using the Hematoxylin & Eosin kit (C0105S, Beyotime) according to the manufacturer’s instructions. For frozen section, after optimal cutting temperature compound (OCT) embedding and sectioning, the Oil Red O kit (C0158S, Beyotime) and Mayer’s hematoxylin (ab220365, abcam) were used to stain triglycerides and the nucleus on frozen crop sections. A digital microscope system was then used to collect images of the crop slices. The Image-pro Plus 6.0 Image analysis system was used to calculate crop thickness. Up to 5 fields of view (more than 200 parts) for each stage were selected from H&E section for statistical analysis. Data on crop epithelial thickness were expressed as the mean ± standard deviation. To quantify the triglyceride content among the ‘lactating’ crops, frozen crop mucosa was ground by mortar and pestle under liquid nitrogen and examined with the Triglyceride assay kit (A110-1-1, Nanjing Jiancheng Bioengineering Institute) according to the manufacturer’s instructions.

Statistical analysis on pigeon hatching behavior or feeding activity was implemented on five pairs of pigeons videotaped over 24-hour using video cameras. The videos were recorded, observed, and the proportion of time in both sexes for each day counted.

### DNA preparation and genome sequencing

To sequence the pigeon genome, we sampled genomic DNA from the pectoral muscle of a healthy male pigeon. DNA was extracted using the DNeasy Blood & Tissue Kit (69506, QIAGEN) according to the instructions. The gDNA integrity was assessed with an Agilent Bioanalyzer 2100 (Agilent Technologies), and the concentration measured by Qubit.

We used two high-throughput platforms (Pacific Bioscience Sequel and Illumina Hiseq X Ten) for pigeon genome sequencing. For PacBio, genomic DNA was fragmented into ~20 kb fragments. SMRT adapters were connected to the DNA to form the SMRTbell library using the SMRTbell Template Prep Kits (100-259-100, PacBio) according to the manufacturer’s protocol, then DNA sequencing was performed on the PacBio Sequel System. For 150 bp paired-end (PE) sequencing, DNA libraries were prepared using the Illumina TruSeq DNA HT Sample Prep Kit (FC-121-2003, Illumina), and sequenced on an Illumina Hiseq X Ten platform.

To improve the integrity of the genome assembly and construct the chromatin architecture of the ‘lactating’ crop, we isolated the mucosa layer of crop to construct the Hi-C libraries. Crop mucosa samples were obtained from female pigeons and homogenized, samples fixed with 2% formaldehyde at room temperature for 30 min. The reaction was stopped using 0.2 mol·L^−1^ glycine. After cell lysis (1 mol·L^−1^ Tris-HCl, 1 mol·L^−1^ NaCl, 10% CA-630 and protease inhibitors) on ice for 15 min, DNA was digested with 200 U DpnII restriction enzyme (R0543S, NEB) at 37°C for 2 hours. After obtaining biotinylated DNA fragments (0.4mM Biotin-14-dATP, 19524–016, invitrogen) and performing proximity ligate (T4 DNA Ligase, L6030-HC-L, Enzymatics), DNA fragments were sheared to 300–500 bp and isolated. Then the Hi-C libraries were amplified for a total of 10 PCR cycles. Genome assembly used Hi-C sequenced libraries as 150 bp paired-end reads on an Illumina Hiseq X Ten platform. To reconstruct the chromatin architecture of the crop during ‘lactation’, Hi-C libraries were sequenced as 100 bp paired-end reads on a BGISEQ-500 platform.

### Library construction of bulk RNA-seq

For annotation of protein-coding genes in pigeon genome, we sampled ten different tissues, including liver, lung, kidney, pectoral muscle, leg muscle, testis, brain, abdominal adipose, crop, and retina, from the male pigeon. To analyze the dynamic transcript profile of the crop during pigeon ‘lactation’, 102 females were selected at one Ceased stage (unpaired pigeons), six incubation stages (Incub 1^st^–16^th^ days) and ten nurturing stages (Nur 1^st^–28^th^ days). Similarly, we selected 42 male pigeons from one Ceased stage, two incubation stages (Incub 13^th^ and 16^th^ days) and four nurturing stages (Nur 1^st^, 4^th^, 16^th^ and 28^th^ days), and six biological replicates for each stage were performed. After the animal was sacrificed, the mucosa layer of the crop was sampled. RNeasy Mini Kit (74004, Qiagen) was used to extract the total RNA for each tissue according to the manufacturer’s instructions. To obtain broader expressed genes on the genomic annotation analysis, we mixed total RNA from different tissues at equal concentrations.

The rRNA depletion protocol was performed as previously described [97]. Briefly, the ribosome-free RNA was obtained using the rRNA Removal Kit (RZH1046, Epicentre). Next, RNA Library Prep Kit (E7420S, NEB) was used to construct the RNA sequencing library. Then, RNA libraries were sequenced as 150 bp paired-end reads on the Illumina Hiseq X Ten platform or 100 bp paired-end reads on a BGISEQ-500 platform for genome annotation analysis or profiling ‘lactating’ crop transcription, respectively.

### Library construction of spatial transcriptome

We performed spatial-transcriptomic library construction using the representative crop tissues, including at the Ceased stage, Incub13^th^ day, and Nur1^st^ day female pigeons, according to the protocol provided by 10X Genomics. 6.5 mm × 6.5 mm × 1 cm crop tissue sections were embedded at the optimal cutting temperature compound (OCT) and stored at −80°C. The samples were then cryogenically sectioned into 15 μm slices and placed within a Visium Spatial slide. After permeabilization and fluorescent cDNA synthesis, fluorescence images were scanned by a digital slice scanner, and the optimal permeabilization time selected by combining fluorescence intensity and RNA diffusion degree. After fragmentation and labeling of the barcodes, Spatial-transcriptomic libraries were sequenced as paired-end reads on the Illumina NovaSeq 6000.

### ChIP-seq library construction

To assess the activities of putative enhancers involved in PEIs, we performed ChIP-seq using antibodies against H3K27ac, with 2 biological replicates for the crop mucosa samples at two representative time points (Ceased stage and Nur 1^st^ day). The ChIP-seq experiments were performed as previously described [98]. Samples were fixed by 1% formaldehyde fixed tissues, and DNA was obtained after cell lysis. DNA was then fragmented to an average fragment size of 200–500 bp with a sonicator. Half of the soluble fragment was stored at −20°C for input DNA and the remaining was used for immunoprecipitation reacting with H3K27ac (ab4729, Abcam) antibodies. Both input and immunoprecipitated ChIP-seq libraries were sequenced on an Illumina HiSeq X Ten platform to generate 150bp paired-end reads.

### Liquid chromatography-high resolution mass spectrometry

LC-MS-based metabolomics analysis was performed on male and female pigeon ‘milk’ at representative time points, including Nur 1^st^ day of 3 female pigeons, and of 9 male pigeons; and Nur 19^th^ day of 9 male pigeons. Pigeon ‘milk’ was homogenized in liquid nitrogen. After addition of 200 μl methanol/water (4:1) pre-cooled at –40°C the samples were immediately vortexed for 15 s, sonicated for 15 min at 20°C and centrifuged for 10 min at 19000 x g. The supernatants were subjected to a second extraction using 200 μl methanol/water (4:1). The combined extracts were evaporated to dryness in a vacuum centrifuge at 30°C, thoroughly reconstituted in 200 μl methanol/water (3:7), and filtered using 0.2 μm PTFE syringe filters. Chromatographic separations were performed on an Acquity UPLC system (Waters) equipped with HSS T3 column (100 x 1.0 mm, particle size 1.8 μm, Waters). After eluting the ions from the column, high-resolution tandem mass spectrometry (Xevo G2-XS QTOF, Waters, UK) was performed in positive or negative mode.

### *De novo* genomic assembly

A total of 86.75 Gb DNA sequencing data (coverage of 63.88×) were obtained from the PacBio Sequel System. After the self-error correction step, error-corrected pre-assembly reads were used to assemble the contigs using the FALCON software according to the Overlap-Layout-Consensus algorithm [99]. The assembled genomic sequences were further polished with Quiver using the PacBio long reads. After this, another round of genome-wide base-level correction was performed with the 84.72 G Illumina 150 bp paired-end (PE) DNA data using the Pilon software [100]. The 213.32 G contacts were generated from the Hi-C library and created as previously reported [101]. After aligning the Hi-C reads to the assembled contig sequences with BWA (v 0.7.15) [102], proximity-guided assembly (PGA) scaffolding was performed using the Lachesis software as previously described [103]. Finally, we obtained a chromosome-level high-quality pigeon genome in which the contigs N50 was 7.79 Mb and the scaffolds N50 was 35.84 Mb.

### Gene annotation

Protein-coding gene prediction using a combined strategy of *ab initio* prediction, homologous comparison, and transcript-based prediction methods. For *ab initio* prediction, the software GenScan [104], Geneid [105], and Augustus [106] were used, while the homology-based method utilized the protein and coding sequences of chicken, duck, human, mouse, turkey, zebra finch from the Ensembl database [107] and aligned them against the pigeon genome using TBLASTN [108] with an E-value cutoff of 1.00×10^−5^. After filtering, all blast hits were concatenated and the gene structure predicted using GeneWise [109]. RNA-seq reads were also aligned to the pigeon genome and used to predict protein-coding regions using TopHat [110] and Trinity [111]. Finally, the results from the three prediction strategies were integrated using EVM [112] with default parameters, this yielding a total of 18,660 protein-coding genes in the pigeon genome.

The functional annotation of protein-coding genes of the pigeon genome was predicted by aligning protein sequences against public databases, specifically SwissProt [113] and KEGG [114] using BLASTP [115, 116] with an E-value cutoff of 1.00×10^−5^. The InterPro database was utilized to annotate protein motifs and domains using the InterproScan [117] tool. Gene Ontology (GO) [118] terms for each protein-coding gene were obtained from the corresponding InterPro entry.

### Analysis of gene expression profile

We used Kallisto [119] to quantify gene expression and obtain TPM expression values by mapping the reads to the predicted coding regions of genes. Differentially expressed genes (|log_2_FC| > 1, FDR < 0.05) were estimated using edgeR (v 3.22.5) [120] based on abundance files generated by Kallisto. MaSigPro (v 3.12) [22] was used to identify the genes with dynamic temporal profiles. TPM values of differentially expressed genes between time points were inputted with MaSigPro and the time series change value was selected when the goodness-of-fit (R^2^) was 0.3. *K*-means clustering was performed to identify stage-specific expression profiles. For the RNA-seq signal graph, we used the number of extracted reads in each window in the BAM file by SamTools (v 1.3.1) [121]. Data visualization was performed with IGV (v 2.3.91) [122].

### Hi-C data processing

For Hi-C data mapping, filtering, and normalization, we used the Juicer pipeline (v 1.5.6), as described in a previous report [101]. The high-quality Hi-C data were mapped to the assembled genome using BWA (v 0.7.15) [102]. Aligned read pairs were distributed to the restriction motif fragment. After filtering duplicates, low-quality alignment read pairs (MAPQ < 30) and intra-fragment read pairs, the Hi-C valid contacts were used to generate intra-chromosomal contract matrices at 5, 20 and 100 kb resolutions. The matrices were normalized using the Knight-Ruiz (KR) matrix balancing algorithm [123] and the Quantile [124] algorithm. We used HiCRep [125] with default parameters to assess the repeatability of the contract matrices.

Compartment A/B identification at 20 kb resolution matrices was implemented using the A-B index value as described previously [126]. Firstly, the ‘prcomp’ function in R was performed on 100 kb resolution contact matrices to generate PC1 vectors of each chromosome per sample, and the Spearman’s correlation between PC1 vectors and genomic characteristics (*i*.*e*., gene density and GC content) calculated. In chromosomes with positive Spearman’s *r*, the bins with positive PC1 vectors were defined as compartment A in low-resolution (*i*.*e*., 100 kb resolution). In contrast, they were instead classified as compartment B. We then measured the A-B index at 20 kb resolution and calculated the median log_2_ distance normalized interaction score with 100 kb resolution compartment A/B. An A-B index was created by subtracting the A and B scores. The bins with positive values (more association with the A compartment) were defined as A at 20 kb resolution, with the remainder classified as compartment B. To identify compartment switch regions, we defined a set of common A/B compartments (all biological replicates exhibiting the same A/B compartment status). Regions with a common A-B status transition between Cease and Nur 1^st^ day were defined as A-B switch.

To identify topologically associated domains (TADs), we combined the directionality index [126, 127] (DI) and the insulation index (IS) [128], and applied them to the 20 kb resolution matrices. The DI was calculated +/- 10 bins from the center of each bin at 20 kb resolution. A hidden Markov model (HMM) was then used to predict the states of DI for the TADs borders. After this, based on the TADs identified by DI, we used minimal IS (along the normalized insulation score vector) to further divide TADs into smaller units. Specific TAD boundaries were identified as previously reported [127]. Due to the high repeatability of TADs in biological repeats, the reads of different biological repeats were pooled together to calculate the ID and identify the specific TAD boundary between time points. Briefly, we calculated the Spearman correlation coefficient of DI +/- 10 bins from the center of the boundary for two samples at adjacent time points. Spearman’s *r* was calculated from 20 randomly selected bins between each adjacent time point sample for random correlation. Each randomization was repeated 1000 times to obtain the random distribution of Spearman correlated coefficients. A specific boundary was defined as those only identified at one-time point and lacking significance compared to the random correlation distribution. Then, we use *D*-score [129] to quantitate intra-TAD strength, a given TAD *D*-score were defined by the ratio of intra-TAD interactions over all interactions for consensus TAD (intra-TAD and inter-TAD). TADs with Student’s *t*-test *P* value < 0.05 and top 5% delta *D*-score value between stages were considered as different strength TADs.

### Promoter-Enhancer interaction (PEI) analysis

To identify PEI at the single gene level, the reads of different biological repeats were pooled together to generate 5 kb resolution contact matrices. The normalized contact matrix was split into the smaller matrix (20 Mb × 20 Mb) with 10 Mb steps of overlapped length, and subsequently analyzed in PSYCHIC [130] algorithms with default parameters to identify over-represented interactions from the promoter region (range from -2 kb to +0.5 kb of transcription start site). We kept the high confidence PEIs with FDR values ≤ 0.05 and interaction distances ≥ 40 kb. To characterize enhancer additivity, we introduced a regulatory potential score (RPS) [64] consisting of the sum of all significant interaction intensities within a given gene, *i*.*e*., ∑n (log_10_In), where ‘In’ indicates the normalized interaction intensity (observed number of contacts minus the expected number of contacts).

### ChIP–seq data analysis

Reads were mapped to the assembled genome with BWA (v 0.7.15) [102], allowing for up to two mismatches. SAMtools (v 1.3.1) [121] was used to remove potential PCR duplicates. H3K27ac peaks regions were identified with MACS2 algorithms [131] to find peaks with a *P* value < 0.05. We defined super-enhancers (SEs) using the standard ROSE algorithms [132, 133]. Briefly, neighboring enhancer elements (within 12.5 kb) were defined by merging H3K27ac ChIP-seq peaks and ranking the H3K27ac signal to identify an inflection point. Enhancers above the inflection point were defined as SE peaks, while the remainder were classified as regular-enhancer peaks. Combined with the putative enhancer regions from PSYCHIC analysis (see above), the interaction regions away from the promoter were divided into inactive-enhancers (IEs), regular-enhancers (REs) or super-enhancers (SEs), depending on whether the distal putative interaction site overlapped with the RE peaks or SE peaks. For data visualization, we calculated the ChIP-seq signal of the whole genome per 1 kb bin using the formula: log_2_(mark FPKM/input FPKM).

### Spatial transcriptome sequencing data processing

We use Space Ranger (v 1.1) to demultiplex reads, align images, identify barcodes, and count the number of genes according to the 10X Genomics spatial gene expression analysis pipelines. Mapping was performed to the assembled genome and coding regions of genes. The H&E-stained photos of tissue sections, slide serials, and capture area information of each sequenced sample were used for gene counting using the “spaceranger counts” command.

We used the Seurat (v 3.2) [134] package in R (v 4.1.3) for downstream analysis. Firstly, the spots with low-quality, those with >25% of mitochondrial gene expression and those with < 15 expressed genes were filtered out. We then used the “SCTransform” function to normalize the expression matrix [135]. For dimensionality reduction, we performed PCA with the top 10 significant components with the “DimHeatmap” and “ElbowPlot” functions. The “FindNeighbors” and “FindClusters” functions were used to identify clusters. The “RunUMAP” and “DimPlot” functions were used to visualize the clusters, and adjustment resolution parameters were applied to make the spot clustering results conform to the crop’s histological characteristics. To evaluate the reliability of the clustering results, we generated a spatial expression distribution heat map for the *ACTG2*, *E-FABP*, and *CSTA* marker genes for muscle cells, spinous cells, and granular cells, respectively, using the “FeaturePlot” function. To assess the transcriptional profile variation between spatial layers and at different time points, we performed a “pseudo-bulking” analysis by integrating the UMI counts for each gene within each cluster. Briefly, we randomly sampled a set of spots from a given cluster for 3 times, which accounted for 50% of spots found in the given cluster, and integrated the counts per UMI and per gene. We used TPM to quantify gene expression.

### Metabolome data processing

To quantify ion abundance in pigeon ‘milk’, we used Progenesis QI to converse data obtained by the mass spectrometer. After the missing values were filtered and supplemented by the *K*-nearest neighbor algorithm, the data were normalized by probabilistic quotient normalization (PQN) [136]. Annotation was based on reference spectra in MassBack and authentic standards. Differentially abundancy metabolites were identified by |log_2_FC| > 0.5, Student’s *t*-test *P*-value < 0.05, and a VIP ≥ 1 (Variable Importance in Projection, which in orthogonal partial least squares discriminant analysis, PLS-DA). MBRole 2.0 [137] was used for pathway analysis of differentially abundant metabolites.

### Comparative genomics analysis

The protein sequences of nine species (pigeon, chicken, duck, goose, peregrine, saker, turkey, zebra finch and green anole) were collected to perform all-against-all alignment using the software BLASTP (v 2.2.26) [115, 116]. The alignment file was then used to infer gene families by the software OrthoMCL (v 1.4) [138]. The lineage-specific genes for each species were extracted from the results of gene families. Gene family expansion and contraction were detected using the software CAFÉ(v 3.0) [139] software. To identify positively selected genes (PSGs), the sequences of single-copy orthologous genes of nine species were first aligned by the software MUSCLE (v 3.8.31), and the ratio of nonsynonymous to synonymous substitutions (K_A_/K_S_, or ω) of the single-copy ortholog genes were calculated. Then, the branch-site model in the software of PAML (v 4.7) [140] was applied to detect PSGs along the pigeon lineage with a likelihood ratio test (LRT) *P*-value < 0.05 and a Bayesian empirical Bayes (BEB) posterior probability greater than 95% for selected sites.

### Functional enrichment analysis and gene set acquisition

To clarify the functions and biological processes of specific pigeon genes, The all-against-all BLASTP tool [115, 116] was applied to determine the orthologs between pigeon and chicken through the similarities of protein sequences.

We used Metascape (http://metascape.org) for functional enrichment analyses [141]. Genes in the pigeon genome were converted to chicken orthologs, and then converted to human orthologs, which were finally used as inputs for the enrichment. Enrichment analysis was performed against all genes in the genome as the background set, with Gene Ontology-biological processes (GO-BP) as the ontology test set.

Gene sets related to crop functions were obtained from Gene Ontology (GO) and Reactome [142]. All human genes were converted to chicken genes, and then converted back to their respective orthologs in pigeons. Fatty acyls biosynthesis, triglyceride biosynthesis and protein synthesis were obtained from Reactome; phospholipid biosynthesis (GO: 0008654), cholesterol biosynthesis (GO: 0006695), Amino acid biosynthesis (GO: 0008652), Fatty acid transport (GO: 0015908) and amino acid transport (GO: 0015802) were obtained from GO.

## Supporting information

S1 Fig Functional gene categories enriched for the time series genes. The temporal expression profiles (left panel) and top 5 most significantly enriched Gene Ontology-biological process terms (right panel) for ten temporal expression cluster.
(TIF)

S2 Fig Expression of rapidly evolving genes in the pigeon genome. **A.** Comparative genomics analysis among pigeon and seven ‘non-lactation’ avian species (duck, chicken, turkey, goose, zebra finch, peregrine, and saker) along with an outgroup (green anole). The study identified 9433 gene families, including 5077 single-copy orthologous genes, 963 expanded and 1704 contracted gene families in pigeon, and 1164 pigeon-specific genes. A phylogenetic tree (left panel) was reconstructed based on the 5077 single-copy orthologs under the p-distances model. The numbers of gene families with specific-expansions and -contractions are indicated at each branch. The bar plot (right panel) is subdivided into different types of orthologous relationships, including single-copy orthologs, multi-copy orthologs in each genome ("N:N"), multi-copy orthologs in one to eight genomes (“N in 1–8 genomes”), and single- or multi-copy groups with genes in two to eight genomes ("0 in 1–7 genomes"). **B.** The number of positive selection or duplicated genes with transcriptional evidence (left panel), and overlap between temporal expression genes and positive selection or duplicated genes (right panel). **C.** The expression profiles of temporal expression genes with positive selection or duplication. **D.** The top 10 most significantly enriched Gene Ontology-biological process (GO-BP) terms for genes with up-regulated (26 positive selected and 221 duplicated genes) or down-regulated (24 positive selected and 526 duplicated genes) expression during the hypertrophy period.
(TIF)

S3 Fig Examples of crop ‘lactation’- and angiogenesis-related genes in female pigeons. **A-G.** Expression levels of representative genes during the breeding stages (left panel). Diagrams of PEIs, H3K27ac signals, transcription levels, and spatial distribution and expression level of the pseudo bulk profile (from top to bottom, right panel). Student’s *t*-test was used to determine significant differences; *P*-values in left panel were calculated for Ceased stage. n.s., *P* ≥ 0.05; * 0.01≤ *P* <0.05; **0.001≤ *P* < 0.01; ***P < 0.001. RPS is regulatory potential score for genes in the PEI plots. Log_2_FC and *P*-value in the transcription level plots were calculated by edgeR based on gene abundance. **H.** Gene expression levels of the angiogenesis gene set collected from the Gene Ontology dataset (GO: 0001525). Wilcoxon rank-sum test *P*-values were calculated for Ceased stage. n.s., *P* ≥ 0.05; * 0.01≤ *P* <0.05; **0.001≤ *P* < 0.01; ***P < 0.001. **I-K.** Examples of angiogenesis-related genes. Student’s *t*-test *P*-values were calculated for Ceased stage. n.s., *P* ≥ 0.05; * 0.01≤ *P* <0.05; **0.001≤ *P* < 0.01; ***P < 0.001.
(TIF)

S4 Fig Spatial transcriptomic analysis on the crop of non- hypertrophied and hypertrophied periods. **A.** The H&E staining of crop sections used in spatial transcriptomic analysis. Zoom-in view (top panel) showing the marked epithelial layers. **B, C. (B)** UMAP and **(C)** Distribution of spatial expression revealed 3 specific clusters represent muscularis externa, inner epithelium, and superficial epithelium layers, respectively. **D-F.** Distribution of spatial expression (top panel), UMAP (bottom panel) of layer-specific marker genes. **G.** Unsupervised hierarchical clustering (upper panel) and Pearson’s *r* matrix (bottom panel) of the ‘pseudo-bulking’ expression profile. The profile was divided by clusters and time points. **H.** Layer-specific marker gene expression in each cluster using ‘pseudo-bulking’ expression profile. **I.** The top 10 most statistically significant Ontology-biological processes (GO-BP) terms of layer specific expressed genes. These genes were defined as those showing at least 2-fold expression differences between layers in ‘pseudo-bulking’ expression profile, and by taking the intersection as each time point.
(TIF)

S1 Table Detailed information of promoter-enhancer interactions (PEIs) identified in pigeon genome. (XLSX)

S1 Video Regurgitation behavior of parent pigeon. (MP4)

S1 Appendix Metabolome analysis of pigeon ‘milk’ based on LC-MS/MS data. (DOCX)

S2 Appendix Morphological phenotype and transcription profile of male pigeon, and RNA-seq data summary. (DOCX)

S3 Appendix *De novo* pigeon genome assembly by short reads sequencing, single-molecule sequencing, and chromatin conformation capture. (DOCX)

S4 Appendix Construction of chromatin 3D architecture based on crop Hi-C data. (DOCX)

S1 Data Numeric data for graphs in Figures. (XLSX)
