# Supplementary figures and images for: Spatio-temporal transcriptome dynamics coordinate rapid transition of core crop functions in ‘lactating’ pigeon
Source: PLoS Genet. 2023 Jun 8;19(6):e1010746. doi: 10.1371/journal.pgen.1010746 (PMC10249823; doi:10.1371/journal.pgen.1010746)

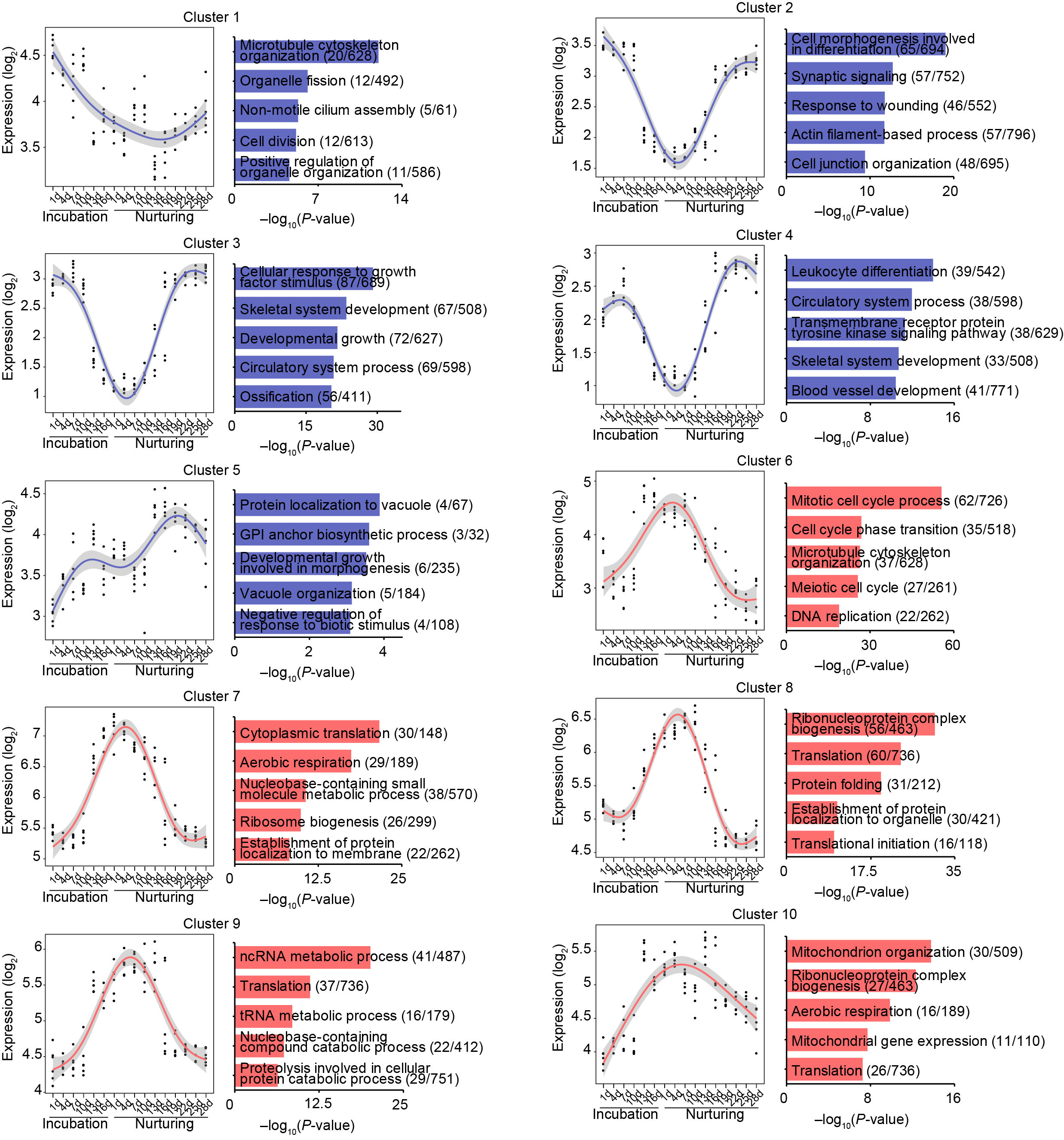

Supplement: S1 Fig — The temporal expression profiles (left panel) and top 5 most significantly enriched Gene Ontology-biological process terms (right panel) for ten temporal expression cluster. (TIF) [file pgen.1010746.s001.tif]

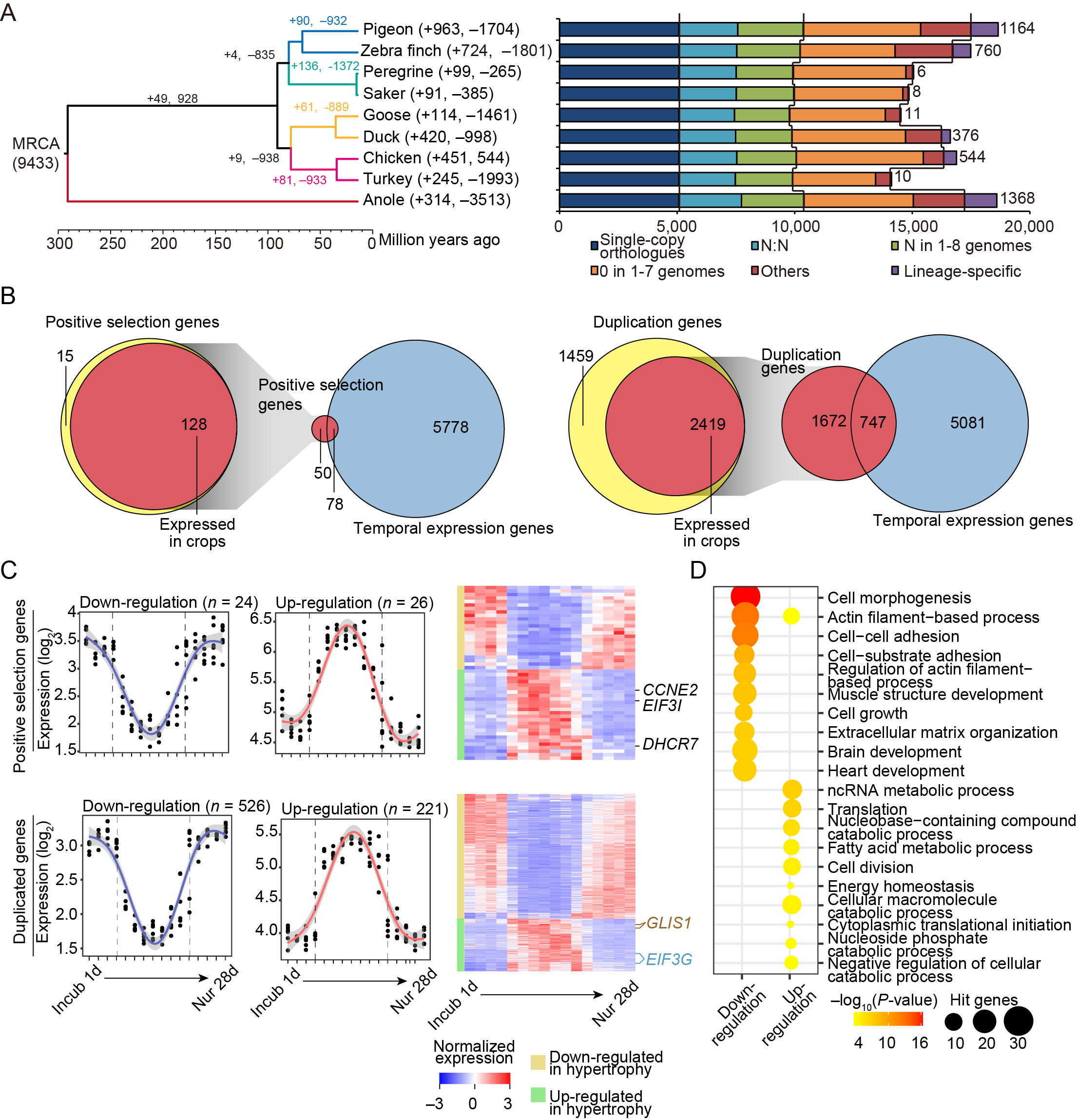

Supplement: S2 Fig — A. Comparative genomics analysis among pigeon and seven ‘non-lactation’ avian species (duck, chicken, turkey, goose, zebra finch, peregrine, and saker) along with an outgroup (green anole). The study identified 9433 gene families, including 5077 single-copy orthologous genes, 963 expanded and 1704 contracted gene families in pigeon, and 1164 pigeon-specific genes. A phylogenetic tree (left panel) was reconstructed based on the 5077 single-copy orthologs under the p-distances model. The numbers of gene families with specific-expansions and -contractions are indicated at each branch. The bar plot (right panel) is subdivided into different types of orthologous relationships, including single-copy orthologs, multi-copy orthologs in each genome ("N:N"), multi-copy orthologs in one to eight genomes (“N in 1–8 genomes”), and single- or multi-copy groups with genes in two to eight genomes ("0 in 1–7 genomes"). B. The number of positive selection or duplicated genes with transcriptional evidence (left panel), and overlap between temporal expression genes and positive selection or duplicated genes (right panel). C. The expression profiles of temporal expression genes with positive selection or duplication. D. The top 10 most significantly enriched Gene Ontology-biological process (GO-BP) terms for genes with up-regulated (26 positive selected and 221 duplicated genes) or down-regulated (24 positive selected and 526 duplicated genes) expression during the hypertrophy period. (TIF) [file pgen.1010746.s002.tif]

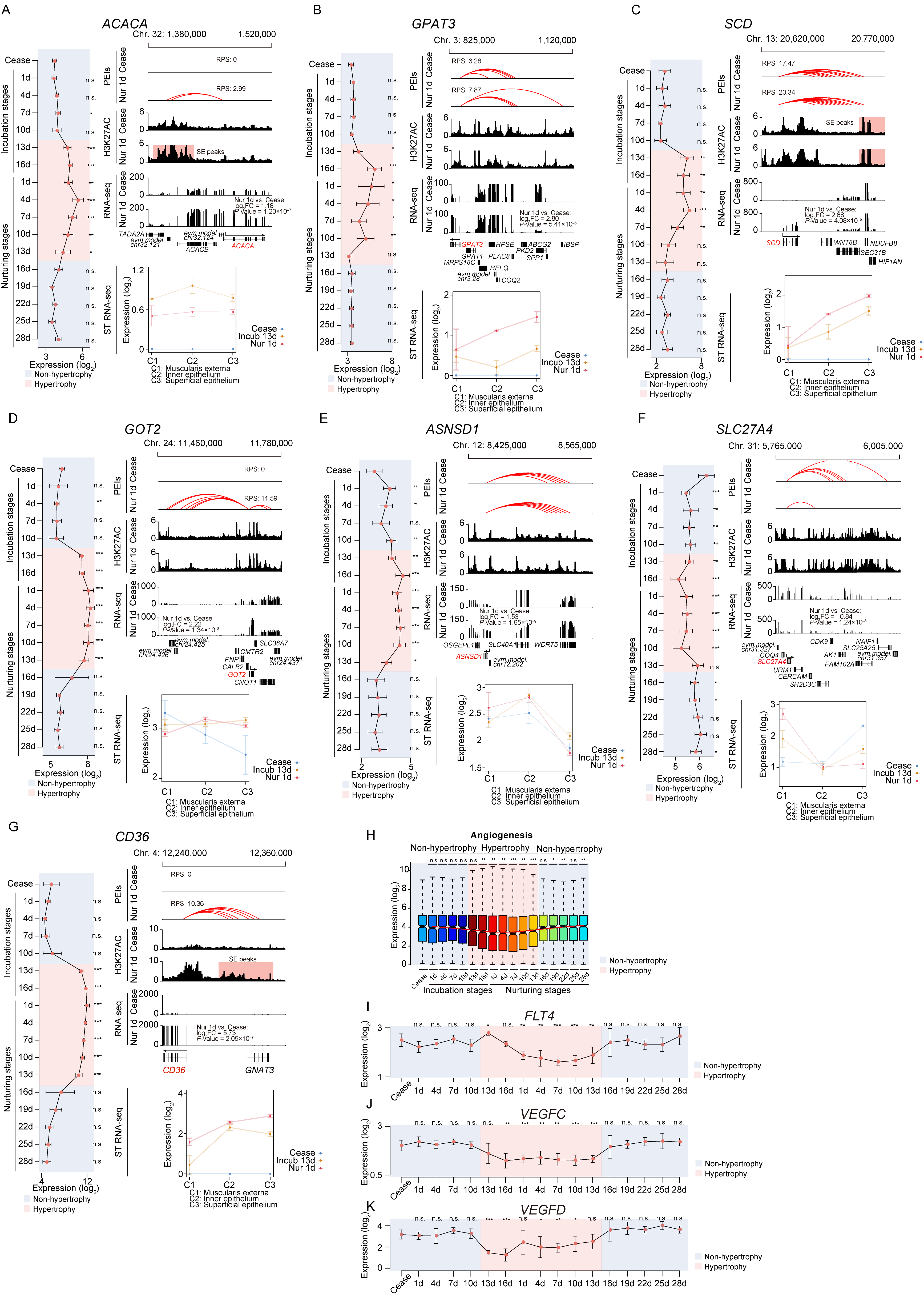

Supplement: S3 Fig — A-G. Expression levels of representative genes during the breeding stages (left panel). Diagrams of PEIs, H3K27ac signals, transcription levels, and spatial distribution and expression level of the pseudo bulk profile (from top to bottom, right panel). Student’s t-test was used to determine significant differences; P-values in left panel were calculated for Ceased stage. n.s., P ≥ 0.05; * 0.01≤ P <0.05; **0.001≤ P < 0.01; ***P < 0.001. RPS is regulatory potential score for genes in the PEI plots. Log2FC and P-value in the transcription level plots were calculated by edgeR based on gene abundance. H. Gene expression levels of the angiogenesis gene set collected from the Gene Ontology dataset (GO: 0001525). Wilcoxon rank-sum test P-values were calculated for Ceased stage. n.s., P ≥ 0.05; * 0.01≤ P <0.05; **0.001≤ P < 0.01; ***P < 0.001. I-K. Examples of angiogenesis-related genes. Student’s t-test P-values were calculated for Ceased stage. n.s., P ≥ 0.05; * 0.01≤ P <0.05; **0.001≤ P < 0.01; ***P < 0.001. (TIF) [file pgen.1010746.s003.tif]

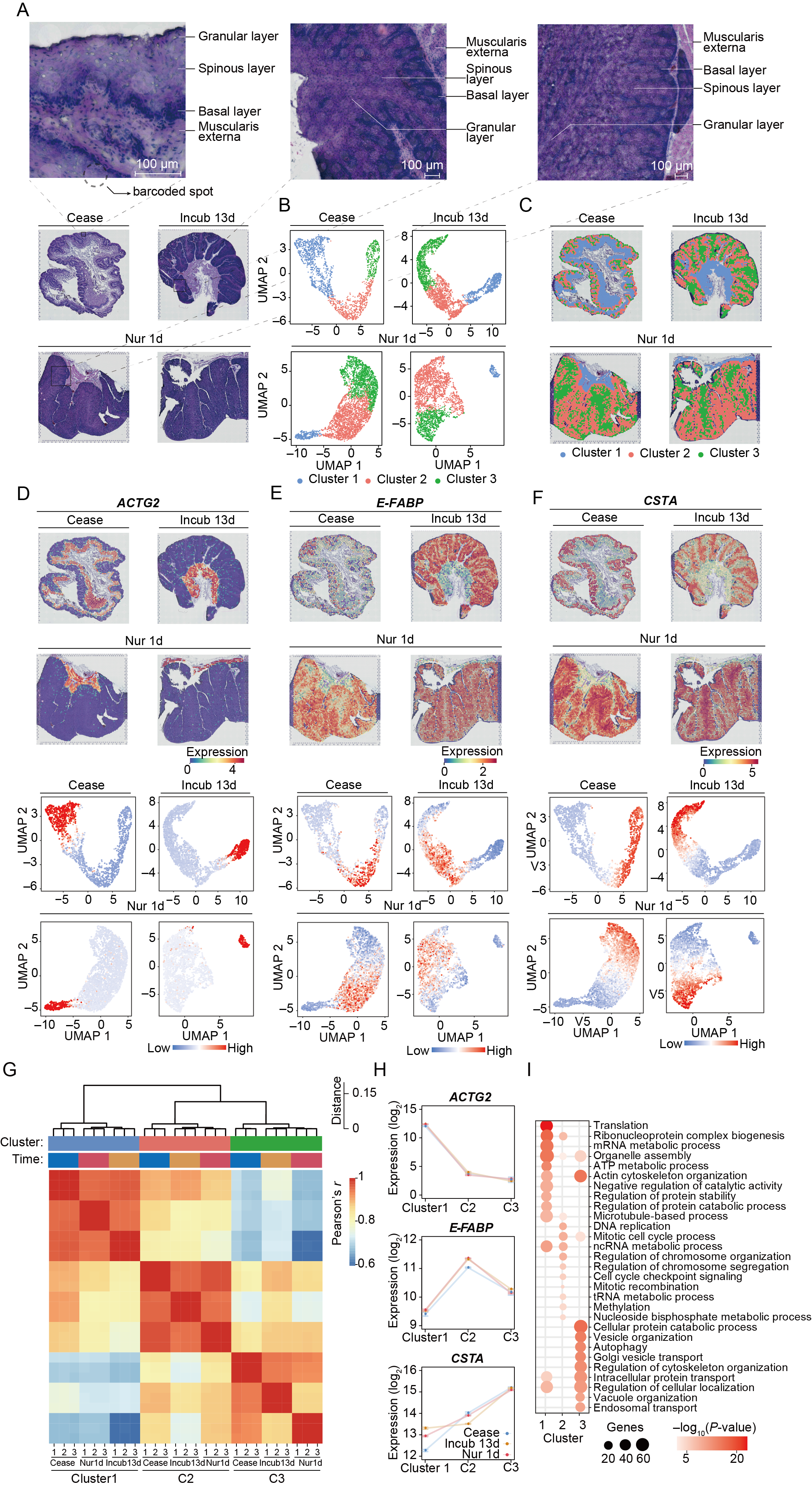

Supplement: S4 Fig — A. The H&E staining of crop sections used in spatial transcriptomic analysis. Zoom-in view (top panel) showing the marked epithelial layers. B, C. (B) UMAP and (C) Distribution of spatial expression revealed 3 specific clusters represent muscularis externa, inner epithelium, and superficial epithelium layers, respectively. D-F. Distribution of spatial expression (top panel), UMAP (bottom panel) of layer-specific marker genes. G. Unsupervised hierarchical clustering (upper panel) and Pearson’s r matrix (bottom panel) of the ‘pseudo-bulking’ expression profile. The profile was divided by clusters and time points. H. Layer-specific marker gene expression in each cluster using ‘pseudo-bulking’ expression profile. I. The top 10 most statistically significant Ontology-biological processes (GO-BP) terms of layer specific expressed genes. These genes were defined as those showing at least 2-fold expression differences between layers in ‘pseudo-bulking’ expression profile, and by taking the intersection as each time point. (TIF) [file pgen.1010746.s004.tif]
