# Supplementary material for: Spatio-temporal transcriptome dynamics coordinate rapid transition of core crop functions in ‘lactating’ pigeon
Source: PLoS Genet. 2023 Jun 8;19(6):e1010746. doi: 10.1371/journal.pgen.1010746 (PMC10249823; doi:10.1371/journal.pgen.1010746)
Supplement: S1 Appendix — (DOCX) [file pgen.1010746.s007.docx]

**S1 Appendix. Metabolome analysis of pigeon ‘milk’ based on LC-MS/MS data.**


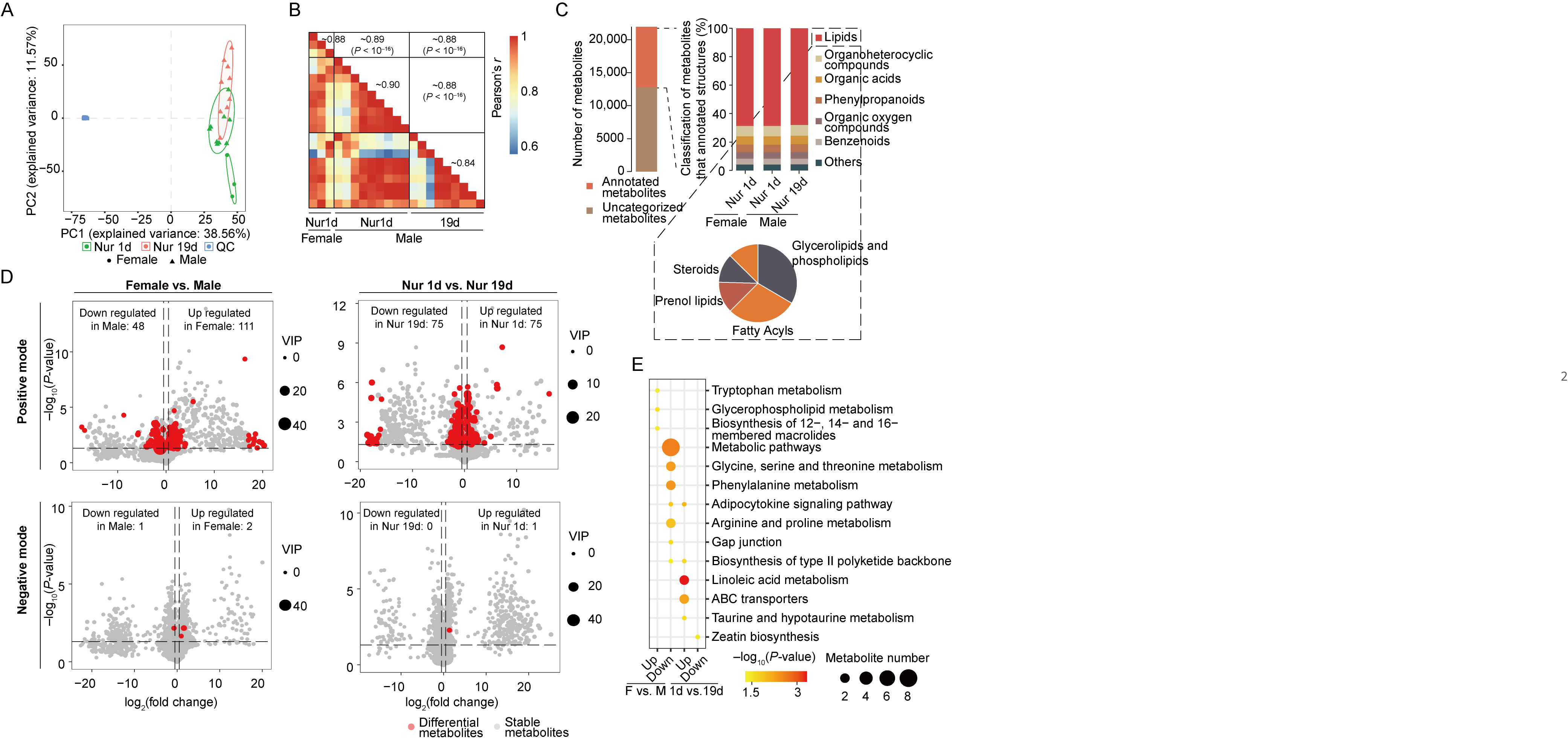


**S1 Appendix. Untargeted metabolome analysis on pigeon ‘milk’. A.** Principal component analysis (PCA) of pigeon ‘milk’ metabolites data. **B.** Pearson correlation coefficient matrix of metabolites data. **C.** Annotation of metabolites (left panel). The composition of metabolites was consistent between the different sexes and feeding time (right panel), and the composition of lipids (bottom panel). These results were consistent with previous studies [1, 2] using chemical methods. **E.** Volcano plot showing different abundant metabolites between different sexes and feeding time. The metabolites with VIP > 1, |log_2_FC| > 0.5 and Student’s *t*-test *P* < 0.05 were classified as differential metabolites. **F.** The statistically significant KEGG pathways of metabolites with differential abundance, which are mainly involved in lipid and amino acid metabolism.

**Reference**

1. Shetty S, Bharathi L, Shenoy K, Hegde SJJoCPB. Biochemical properties of pigeon milk and its effect on growth. 1992;162(7):632-636.

2. Hegde SN. Composition of pigeon milk and its effect on growth in chicks. Indian journal of experimental biology. 1973;11(3):238-239.
