# Supplementary material for: Spatio-temporal transcriptome dynamics coordinate rapid transition of core crop functions in ‘lactating’ pigeon
Source: PLoS Genet. 2023 Jun 8;19(6):e1010746. doi: 10.1371/journal.pgen.1010746 (PMC10249823; doi:10.1371/journal.pgen.1010746)
Supplement: S2 Appendix — (DOCX) [file pgen.1010746.s008.docx]

**S2 Appendix. Morphological phenotype and transcription profile of male pigeon****, and RNA-seq data summary.**

**S2-I Appendix:**

**
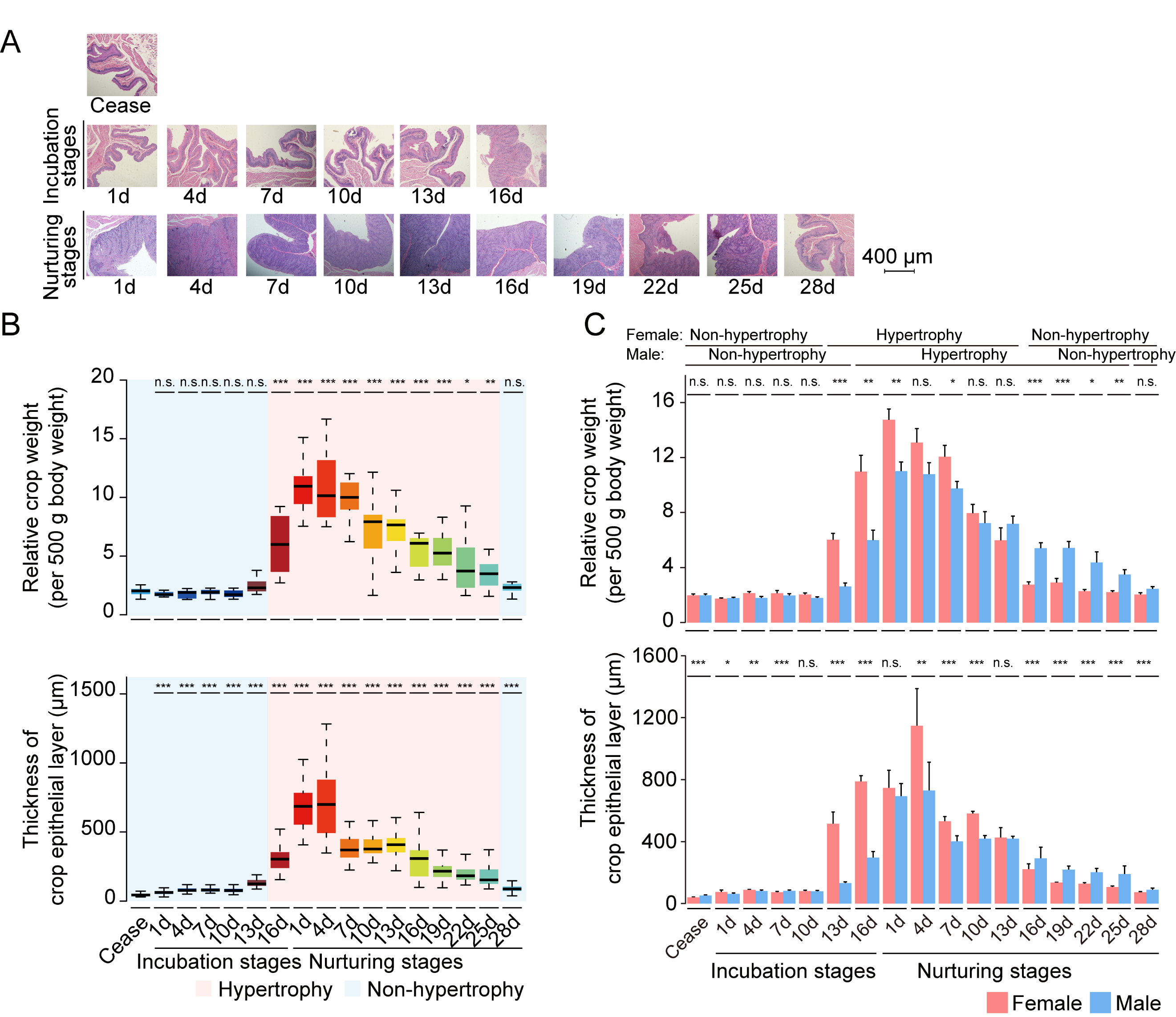
**

**S2-I Appendix.** **Morphological changes in male pigeons during breeding stages. A.** H&E staining of crop paraffin sections in male pigeons. **B.** Crop weight per 500 g of body weight (top panel) and thickness of epithelium section (bottom panel) in male pigeons. Student’s *t*-test *P*-values were calculated with Cease. n.s., *P* ≥ 0.05; * 0.01< *P* <0.05; **0.001< *P* < 0.01; ****P* < 0.001. **C.** Comparison of crop weight (top panel) and epithelium thickness (bottom panel) between female and male pigeon crops. Student’s *t*-test *P*-values were calculated between the sexes within the same time point. n.s., *P* ≥ 0.05; * 0.01≤ *P* <0.05; **0.001≤ *P* < 0.01; ****P* < 0.001.

**S2-II Appendix:**

**
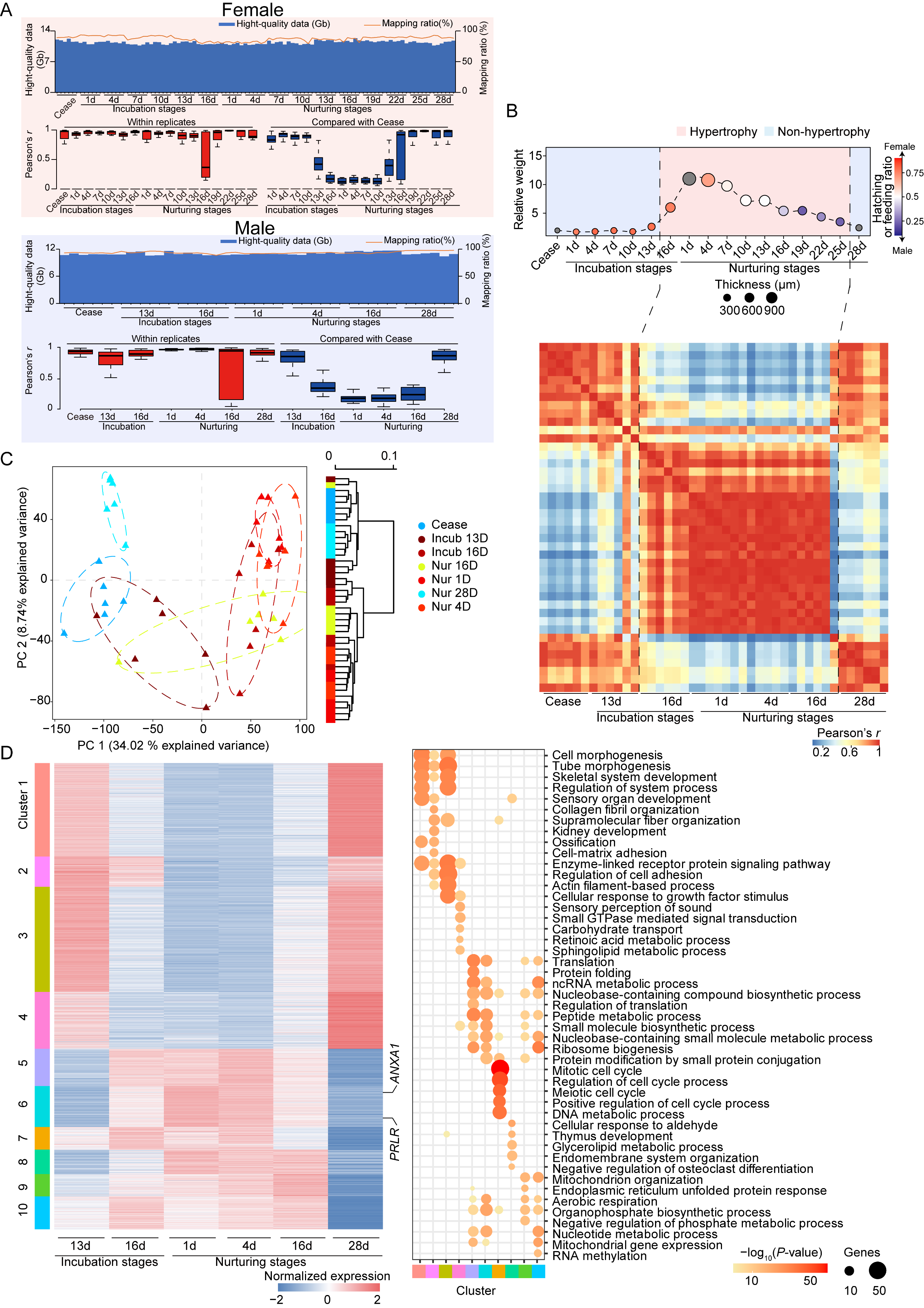
**

**S2-II Appendix. Data summary of RNA-seq and transcription profile of male pigeon. A.** Data summary of RNA-seq (top panel) and similarities of gene expression for female and male crops within replicates and compare with Ceased stage (bottom panel). **B.** Crop phenotypes (top panel) and Pearson correlation coefficient matrix (bottom panel) for male pigeon gene expression profiles. Consistent with the phenotypic data, gene expression patterns show obvious differences between non-hypertrophied and hypertrophied crop. **C.** Unsupervised hierarchical clustering (top panel) and principal component analysis (PCA, bottom panel) of male pigeon gene expression. The dashed line indicates the minimum volume ellipse of each biological repetition. **D.** Ten stage-specific expression clusters are revealed by *k*-means clustering (left panel). The top 5 most statistically significant Gene Ontology-biological process (GO-BP) terms for genes in each cluster (right panel). Gene expression was normalized by *Z*-score in the heat map.

**S2-III Appendix:**


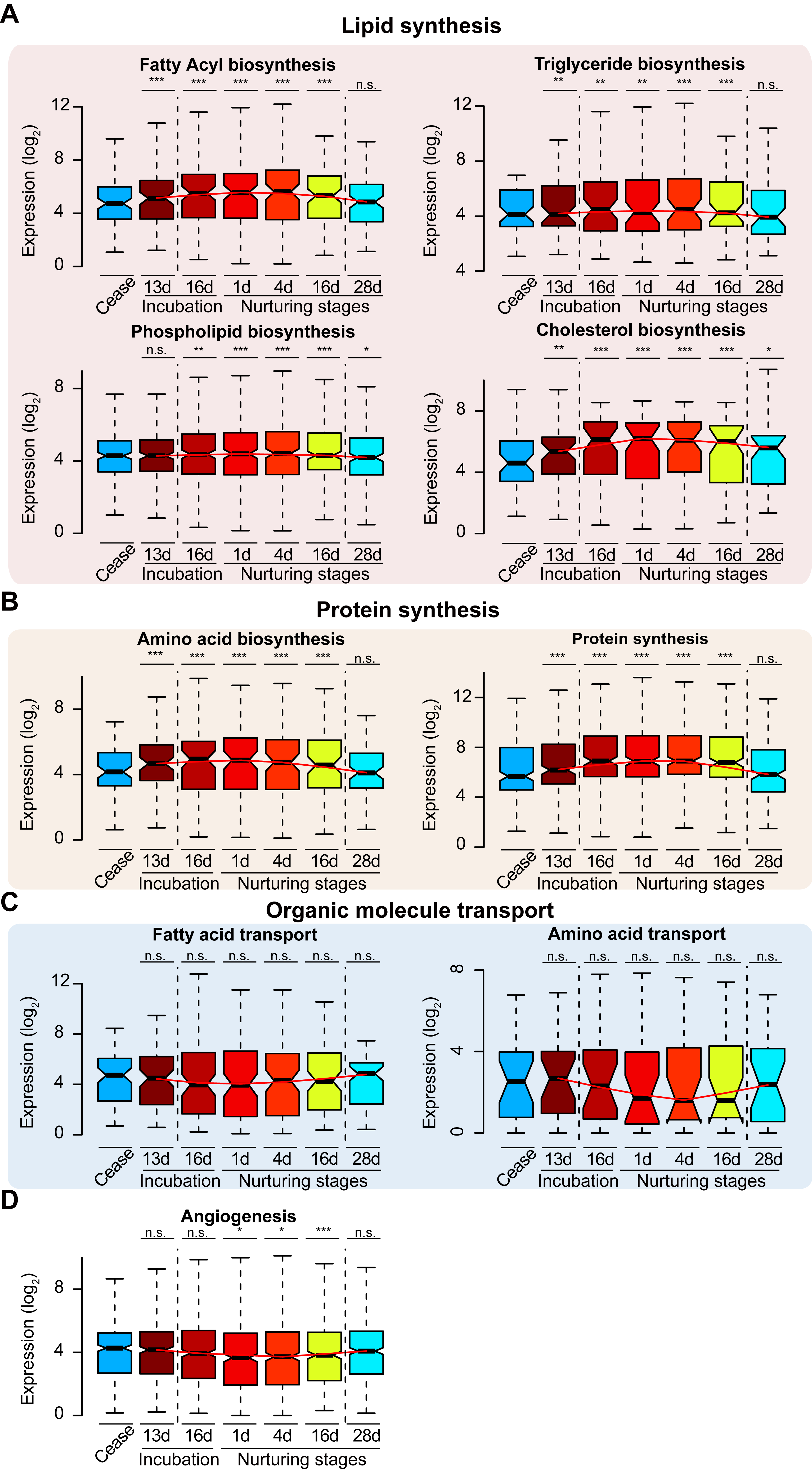


**S2-III Appendix. Expression levels of ‘lactation’-related genes in male pigeons.**

**A-D.** Gene expression levels of eight ‘lactation’-related genes involved in (**A)** lipid synthesis, (**B)** protein synthesis, (**C)** organic molecule transport, and (**D)** angiogenesis identified by GO or Reactome enrichment analyses. Wilcoxon rank-sum test *P*-values were calculated with Ceased stage. n.s., *P* ≥ 0.05; * 0.01< *P* <0.05; **0.001< *P* < 0.01; ****P* < 0.001.
