# Supplementary material for: Spatio-temporal transcriptome dynamics coordinate rapid transition of core crop functions in ‘lactating’ pigeon
Source: PLoS Genet. 2023 Jun 8;19(6):e1010746. doi: 10.1371/journal.pgen.1010746 (PMC10249823; doi:10.1371/journal.pgen.1010746)
Supplement: S3 Appendix — (DOCX) [file pgen.1010746.s009.docx]

**S3 Appendix. *De novo* pigeon genome assembly by short reads sequencing, single-molecule sequencing, and chromatin conformation capture.**

**S3-I Appendix:**

**
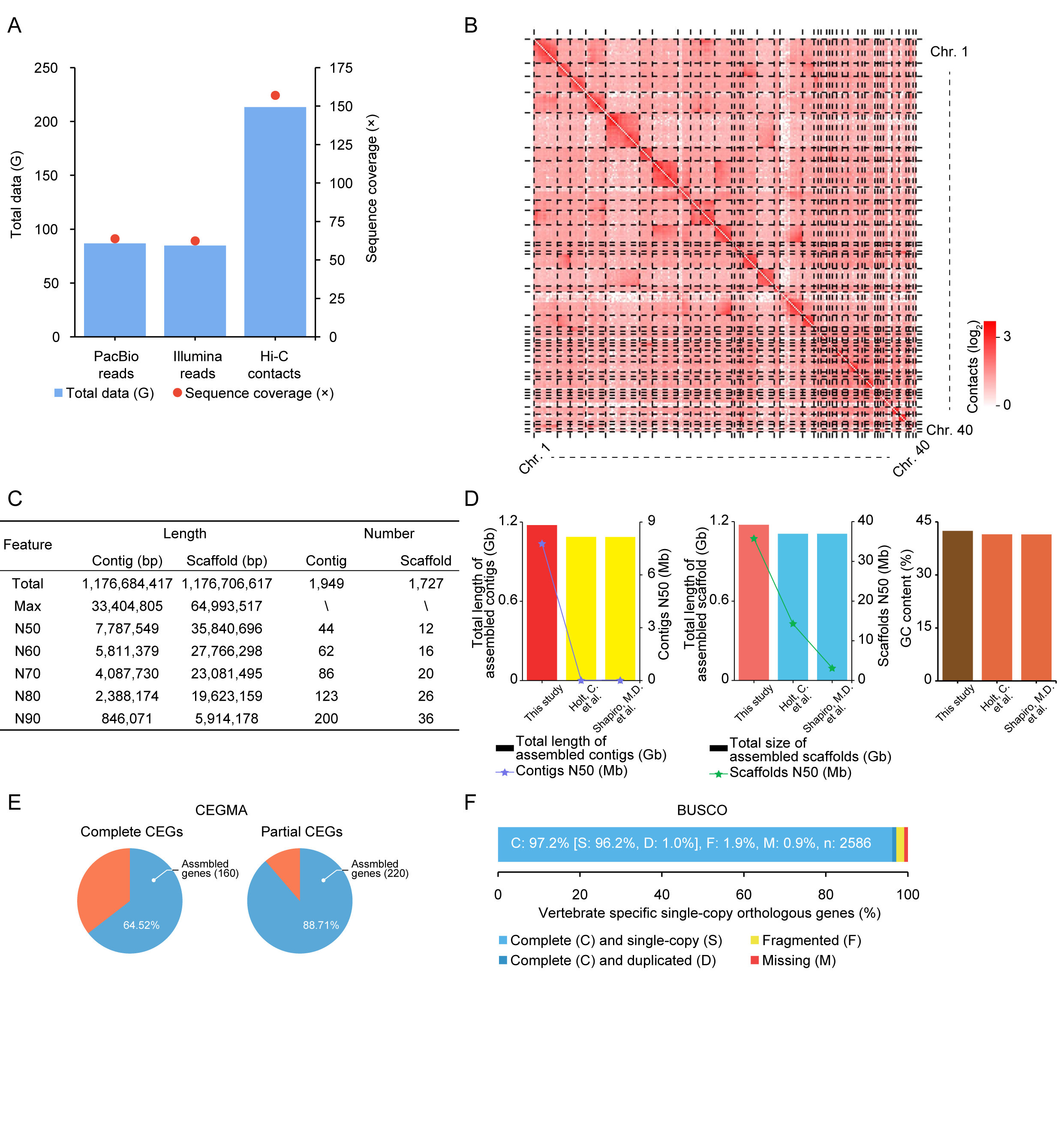
**

**S3-I Appendix. *De novo* assembly of the pigeon genome. A.** Summary of genome sequencing data for pigeon. *De novo* assembly was performed on the White King pigeon genome based on a combined strategy using PacBio, Illumina and Hi-C sequencing data assisted assembly. **B.** Heatmap of the density of Hi-C interactions between pseudo-chromosome. After performing genome assembly based on long reads data and correction of short reads data, Hi-C data were used for obtaining a high-quality pigeon reference genome at the chromosomal level by contigs clustering, sequencing, and orienting. **C.** Summary of the genome assembly. The genome size was estimated at 1,176,706,617 bp. The total length of the first 44 contigs accounted for 50% of the entire genome, indicating the high-quality of the assembled genome. **D.** Comparing the features of the new pigeon genome to previous versions [1, 2]. The quality of this assembly improved dramatically, as shown by contig and scaffold N50 increase to, respectively, more than 290- and 10-fold compared to the previous version [1]. **E.** The CEGMA (Core Eukaryotic Genes Mapping Approach) was used to evaluation the integrity of the assembled genome. A total of 220 genes were assembled from 248 Core Eukaryotic Genes (accounting for 88.71%) in this study. **F.** The BUSCO (Benchmarking Universal Single-Copy Orthologs) was used to evaluate the integrity of the assembled genome. 97.2% of complete single-copy genes were assembled from 2586 lineal homologous single-copy genes. C, S, D, F, M, and n in the stacked bar chart represent complete BUSCOs, complete and single-copy BUSCOs, complete and duplicated BUSCOs, fragmented BUSCOs, missing BUSCOs and total BUSCO groups searched, respectively.

**S3-II Appendix****:**

**
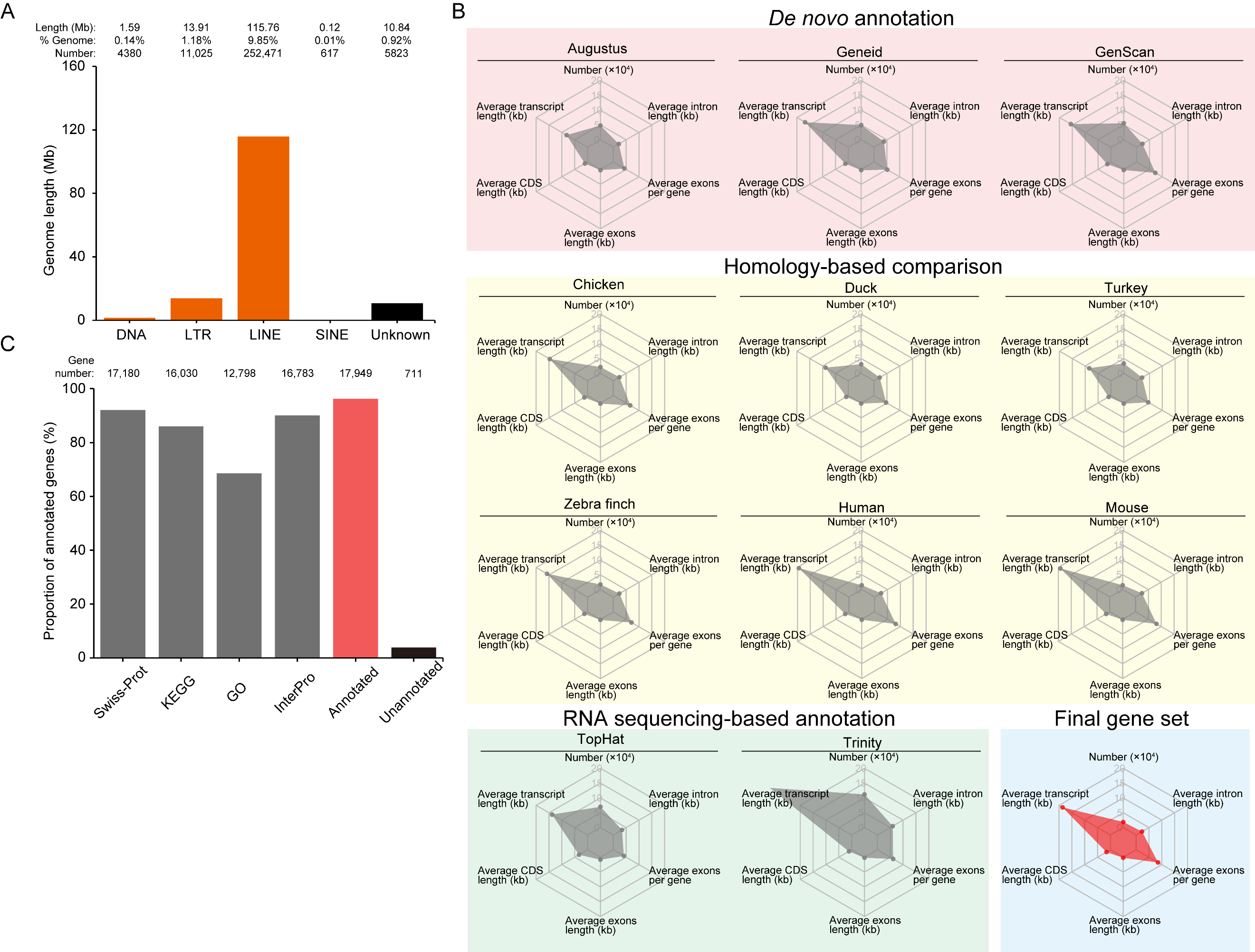
S3-II Appendix FigS3. Annotation of pigeon genome. A.** Repeat elements present in the pigeon genome. Repeat elements were predicted using homology-based comparison and *ab initio* prediction methods. For *ab initio* repeat annotation, LTR-finder [3], RepeatScout [4], and RepeatModeler (<http://www.repeatmasker.org/RepeatModeler/>) were used to construct a *de novo* repetitive element database. The RepeatMasker [5] and RepeatModeler were used to annotate repeat elements with the database. After this, RepeatMasker and RepeatProteinMask [6] were used for integrate repeat element types by searching against the Repbase database [7]. A total of 144.31 Mb of repetitive elements were identified in the pigeon genome, accounting for 12.27% of the whole genome length. This included long terminal repeat elements (LTR, 13.91 Mb, accounting for 1.18% of the total genome), long interspersed nuclear elements (LINE, 115.76 Mb or 9.85%), and short interspersed nuclear elements (SINE, 0.12 Mb or 0.01%). Importantly, the improvement in genome quality promoted the identification of additional repeat elements compared to previous versions [1, 2]. **B.** General statistics of predicted protein-coding genes. The transcripts were annotated using homology-based, *ab initio*-based and RNA sequencing (RNA-seq)-based prediction methods. GenScan [8], Geneid [9], and Augustus [10] were used for *ab initio*–based predictions. *Gallus gallus* (GCF 000002315.6), *Anas platyrhynchos* (GCF 003850225.1), *Meleagris gallopavo* (GCF 000146605.3), *Taeniopygia guttata* (GCF 003957565.1), *Homo sapiens* (GCF 000001405.39), and *Mus musculus* (GCF 000001635.26) were used for homology-based annotation. Liver, lung, kidney, pectoral muscle, leg muscle, testis, brain, abdominal adipose, crop, and retina were pooled to construct the RNA-seq library and used to annotate the protein-coding regions of the assembled genome using TopHat [11] and Trinity [12]. A total of 18,660 protein-coding genes were obtained by EVM [13] integrate. The average transcript length was 18,607 bp, the average CDS length was 1503 bp, the average exon number per gene was 8.5, the average exon length was 176 bp, and the average intron length was 2280 bp. **C.** Functional annotation of annotated gene in assembled pigeon genome. Functional annotation was obtained by aligning protein sequences against public databases, including SwissProt (http://www.gpmaw.com/html/swiss-prot.html), KEGG (http://www.geno me.jp/kegg/) using BLASTP [14] with an E-value cutoff of 1.00×10^-5^. The InterPro database was utilized to annotate protein motifs and domains with the InterproScan tool. Gene Ontology (GO) terms for each protein-coding gene were obtained from the corresponding InterPro entry. A total of 17,949 protein-coding genes (96.19%) were successfully annotated by at least one public database.

**Reference**

1. Shapiro MD, Kronenberg Z, Li C, Domyan ET, Pan H, Campbell M, et al. Genomic diversity and evolution of the head crest in the rock pigeon. Science. 2013;339(6123):1063-1067.

2. Holt C, Campbell M, Keays DA, Edelman N, Kapusta A, Maclary E, et al. Improved genome assembly and annotation for the rock pigeon (*Columba livia*). G3-Genes Genom Genet. 2018;8(5):1391-1398.

3. Benson G. Tandem repeats finder: a program to analyze DNA sequences. Nucleic Acids Res. 1999;27(2):573-580.

4. Price AL, Jones NC, Pevzner PA. *De novo* identification of repeat families in large genomes. Bioinformatics. 2005;21(1):i351-i358.

5. Tarailo-Graovac M, Chen N. Using RepeatMasker to identify repetitive elements in genomic sequences. Curr Protoc Bioinformatics. 2009;25:4-10.

6. Allred DB, Cheng A, Sarikaya M, Baneyx F, Schwartz DT. Three-dimensional architecture of inorganic nanoarrays electrodeposited through a surface-layer protein mask. Nano Lett. 2008;8(5):1434-1438.

7. Bao W, Kojima KK, Kohany O. Repbase update, a database of repetitive elements in eukaryotic genomes. Mob DNA. 2015;6:11.

8. Burge C, Karlin S. Prediction of complete gene structures in human genomic DNA. J Mol Biol. 1997;268(1):78-94.

9. Alioto T, Blanco E, Parra G, Guigó R. Using geneid to identify genes. Curr Protoc Bioinformatics. 2018;64(1):e56.

10. Stanke M, Steinkamp R, Waack S, Morgenstern B. AUGUSTUS: a web server for gene finding in eukaryotes. Nucleic Acids Res. 2004;32:W309-W312.

11. Trapnell C, Pachter L, Salzberg SL. TopHat: discovering splice junctions with RNA-Seq. Bioinformatics. 2009;25(9):1105-1111.

12. Grabherr MG, Haas BJ, Yassour M, Levin JZ, Thompson DA, Amit I, et al. Full-length transcriptome assembly from RNA-Seq data without a reference genome. Nat Biotechnol. 2011;29(7):644-652.

13. Haas BJ, Salzberg SL, Zhu W, Pertea M, Allen JE, Orvis J, et al. Automated eukaryotic gene structure annotation using EVidenceModeler and the program to assemble spliced alignments. Genome Biol. 2008;9(1):R7.

14. Kent WJ. BLAT--the BLAST-like alignment tool. Genome Res. 2002;12(4):656-664.
