# Supplementary material for: Spatio-temporal transcriptome dynamics coordinate rapid transition of core crop functions in ‘lactating’ pigeon
Source: PLoS Genet. 2023 Jun 8;19(6):e1010746. doi: 10.1371/journal.pgen.1010746 (PMC10249823; doi:10.1371/journal.pgen.1010746)
Supplement: S4 Appendix — (DOCX) [file pgen.1010746.s010.docx]

**S4 Appendix. Construction of chromatin 3D architecture based on crop Hi-C data.**

**S4-I Appendix:**


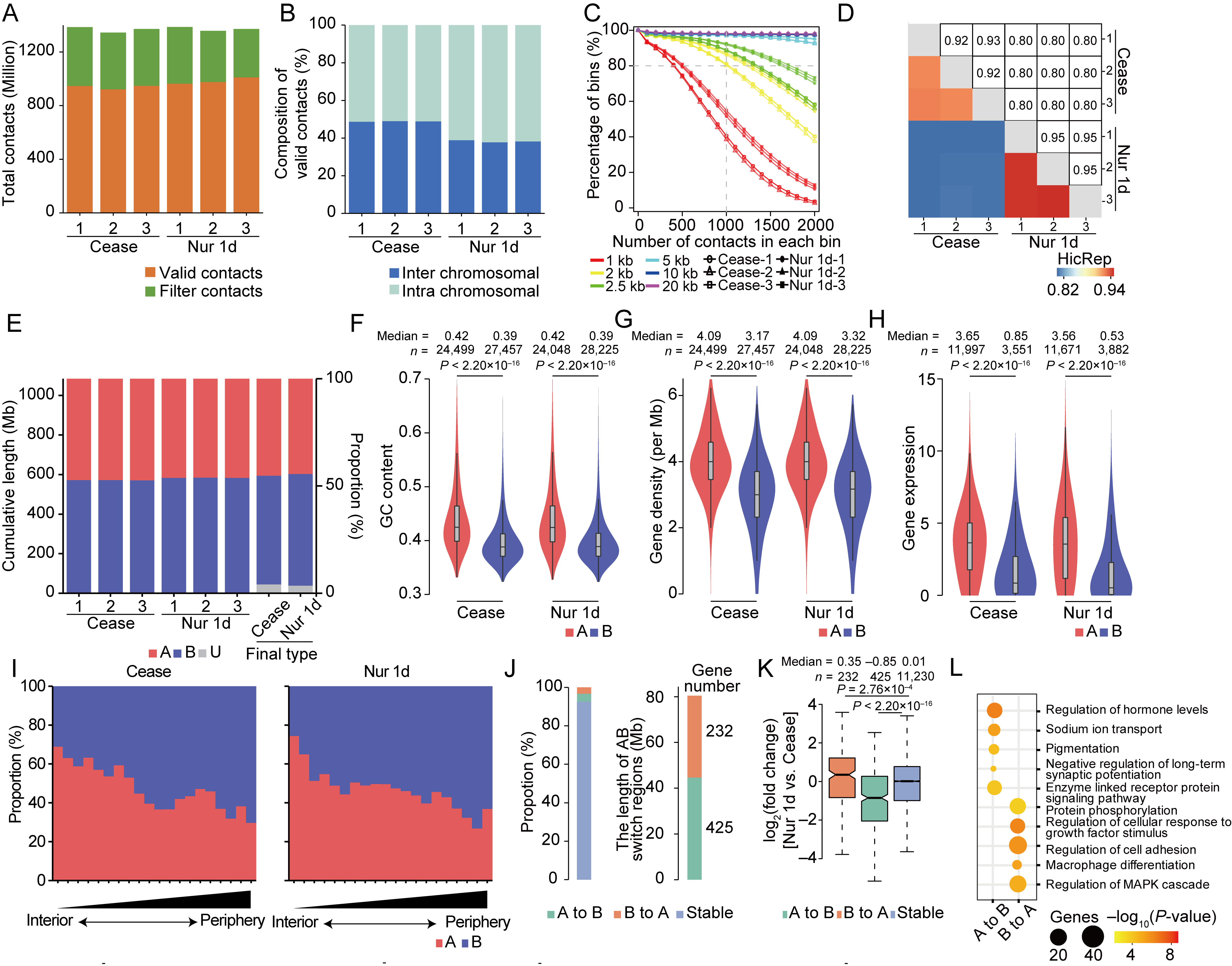


**S4-I Appendix. Hi-C data summary and the genomic features of compartmentalization. A.** Data summary of *in situ* Hi-C. **B.** The proportion of intra- and inter-chromosomal contacts in the valid contacts. **C.** Resolution evaluation of intrachromosomal contact matrices. **D.** Correlation coefficient matrix of HiCRep. **E.** Length and proportions of whole genomic compartmentalization. At 20 kb resolution we identified fewer A (46.12-47.32% of the genome) than B (52.68-53.88% of the genome) compartment regions. The replicates with the same A/B type were defined as final types. **F-I.** Genomic features of A/B compartment, including (**F**) G-C content, (**G**) gene density, (**H**) gene expression and (**I**) Percentage of graph showing trends in the arrangement of the A/B compartments from the interior to the periphery of the nucleus. The nucleus is equally divided into 20 shells based on the relative distance to the nuclear center. As expected, A compartment regions are GC-rich, transcript rich and actively transcribed, and preferentially locate to the interior of the nucleus. In contrast, B compartment regions are GC-poor, transcript-sparse and poorly transcribed, and preferentially localized to the nuclear periphery. **J.** Proportion and length of AB switch regions (~80.44 Mb, 7.42 % of genome), Genes embedded in switched regions are show at the left panel. **K.** Expression changes of genes that located in AB switch regions. *P*-values were calculated using a Wilcoxon-test. Regions with B to A switching were accompanied by an increased in genes expression, with opposite patterns observed in A to B switching regions. **L.** The top 5 statistically significant Gene Ontology-biological processes (GO-BP) of genes embedded in switch regions.

**S4-II Appendix:**


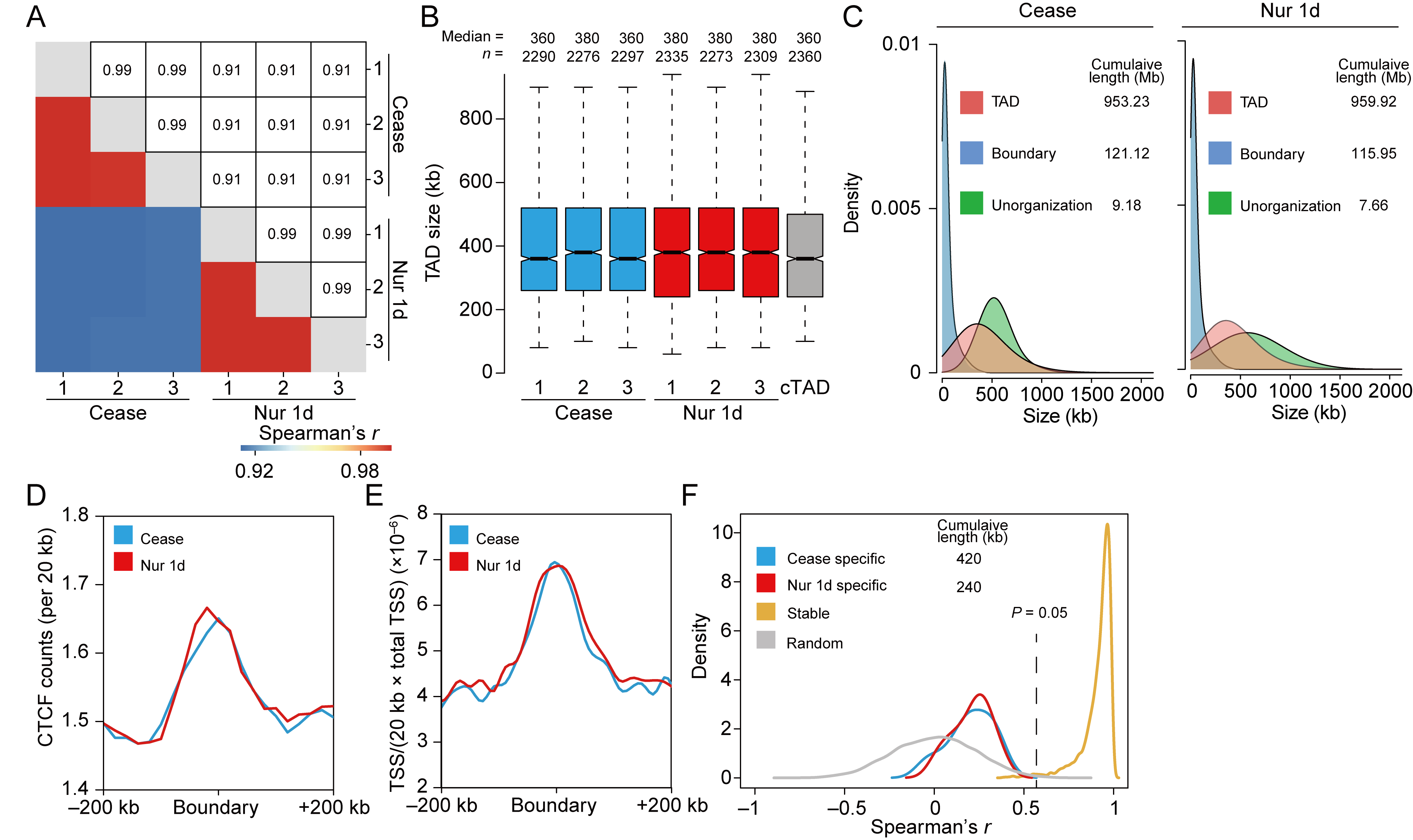


**S4-II Appendix. Inherent features of TAD boundaries. A.** Spearman’s correlation matrix of whole genome insulation score (IS) between Ceased stage and Nur 1d. These results exhibited high reproducibility across biological replicates (Spearman’s *r* > 0.99). **B, C.** (**B**) TADs size and (**C**) density in the crop. The genome was divided into TAD boundaries (median size ≈ 20 kb), TADs (median size ≈ 380 kb) and unorganized regions (median size ≈ 540 kb) at 20 kb resolution. In total, we identified ~2297 TADs (TAD size ~373.34 kb, occupying ~88.13% genome). The cTAD in (**B**) represent consensus TADs during the breeding stage. **D.** Enrichment of CTCF and **E.** transcriptional start site in TAD boundaries. The CTCF motif was identified using the FIMO software based on the JASPAR CORE 2016 vertebrate database. **F.** Density plot of the Spearman’s *r* of directionality indexes between Ceased stage Nur 1d in TAD boundary regions (± 200 kb). Randomly Spearman’s *r* was repeated 1000 times to obtain the random distribution of Spearman correlated coefficients, and showed as a grey line. A specific boundary was defined as those only identified at one-time point and lacking significance compared to the random correlation distribution. A total of 660 kb boundaries were shifted between Ceased stage and Nur 1d.

**S4-III** **Appendix:**


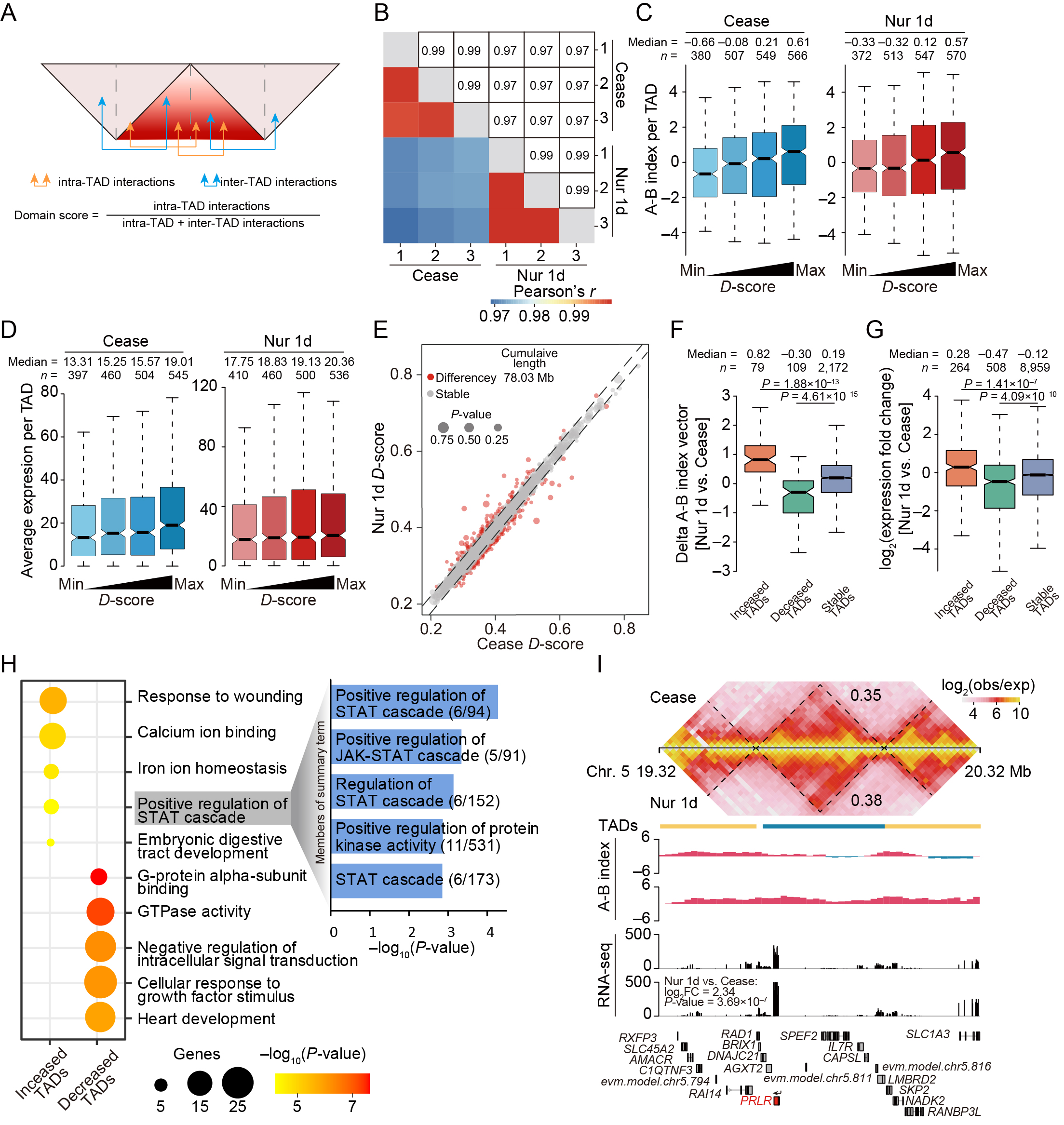


**S4-III Appendix. Inherent features of intra-TAD interactions. A.** The intra-TAD interactions are quantified by Domain-score (*D*-score[1]). **B.** The Pearson’s correlation matrix of TAD *D*-score between Ceased stage and Nur 1d. These results exhibited high reproducibility across biological replicates (Pearson’s *r* > 0.99). **C, D.** (**C**) Average A-B index of TADs and (**D**) the expression levels of genes embedded in the TADs. TADs were divided into four percentiles according *D*-score value percentile. High *D*-score TADs shown high compartmentalization activity and high expression level of gene embedded in TADs. Smaller TADs (TAD size < 200 kb) and transcript-sparse TADs (gene < 2) were filtered. **E.** Dot plot of *D*-score. TADs with Student’s *t*-test *P* value < 0.05 and top 5% delta *D*-score values between Ceased stage and Nur 1d were considered as differential TADs. A total of 78.03 Mb TADs (*n* = 188) were variated with *D*-score between Ceased stage and Nur 1d. **F, G.** (**F**) A-B index changes and (**G**) expression level changes of gene embedded in differential TADs. **H.** The top 5 statistically significant Gene Ontology-biological processes (GO-BP) of genes embedded in differential TADs. **I.** Representative TAD with differential D-score between Cease and Nur 1d. Heatmap indicated the Hi-C contact matrixes, D-score value of the TAD was marked (top panels). the tracks indicated the A-B index (middle panels) and RNA-seq signal (bottom panels). the gene structures are indicated below the tracks, and the transcription direction was indicated black arrows. log_2_(fold change) and *P*-value of gene expression change were calculated by edgeR.

**S4-IV Appendix:**


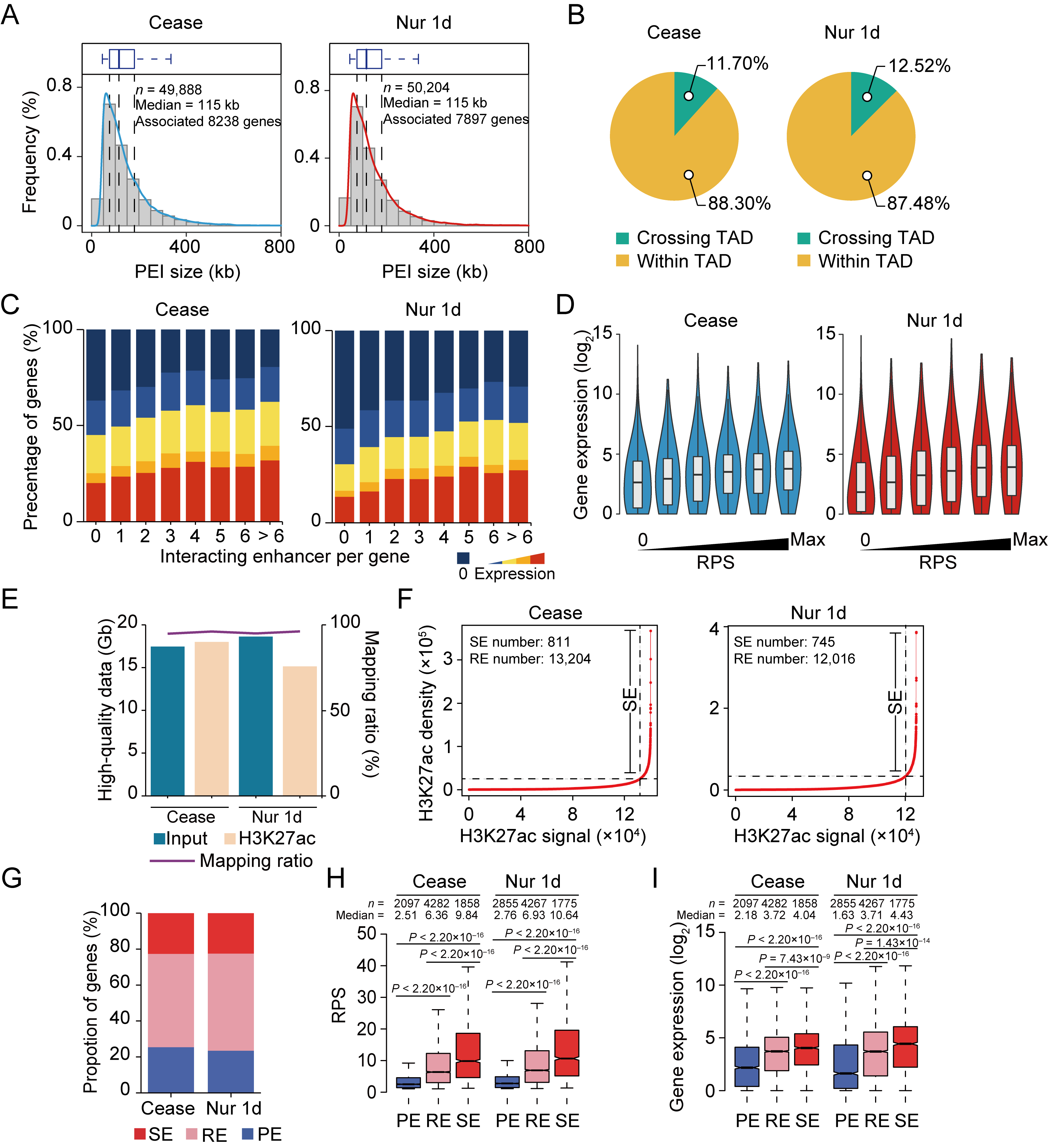


**S4-IV Appendix. The characterization of promoter-enhancer interactions. A.** Distribution of PEIs length. In the boxplot, the internal line indicates the median, the box limits indicate the upper and lower quartiles and the whiskers extend to the 1.5 IQR. We identified ~ 50,046 promoter-enhancer interactions (PEIs, median size of ~115 kb), associated with ~8068 gene promoters. **B.** Proportion of PEIs crossing the TAD boundary. **C.** Percentage of genes from each expression category interacting with different numbers of enhancers (from 0 to >6). As expected, the number of enhancer interactions increases with genes expression levels. **D.** The Regulatory Potential Score (RPS) of different genes are associated with expression levels. Genes were divided into 0 and five percentiles according to RPS, and their respective expression distribution is shown in the violin and box plots. **E.** Data summary of ChIP-seq. **F.** Determining enhancer activity by analyzing H3K27ac signal distribution using the ROSE algorithm. The highly clustered peaks were integrated into the super-enhancer cluster (SEs), while the remainder was classified as regular-enhancers (REs). **G.** Proportion of genes interacting with super-enhancers (SEs), regular-enhancers (REs), and poised-enhancers (PEs). **H.** RPS and **I** expression levels of the gene interaction with different enhancer activities. As expected, ~22.52% of PEI-associated genes interacting with SEs show higher RPS and transcript level than those interacting with REs (~53.01%) and or PEs (~24.47%)

**S4-V Appendix:**


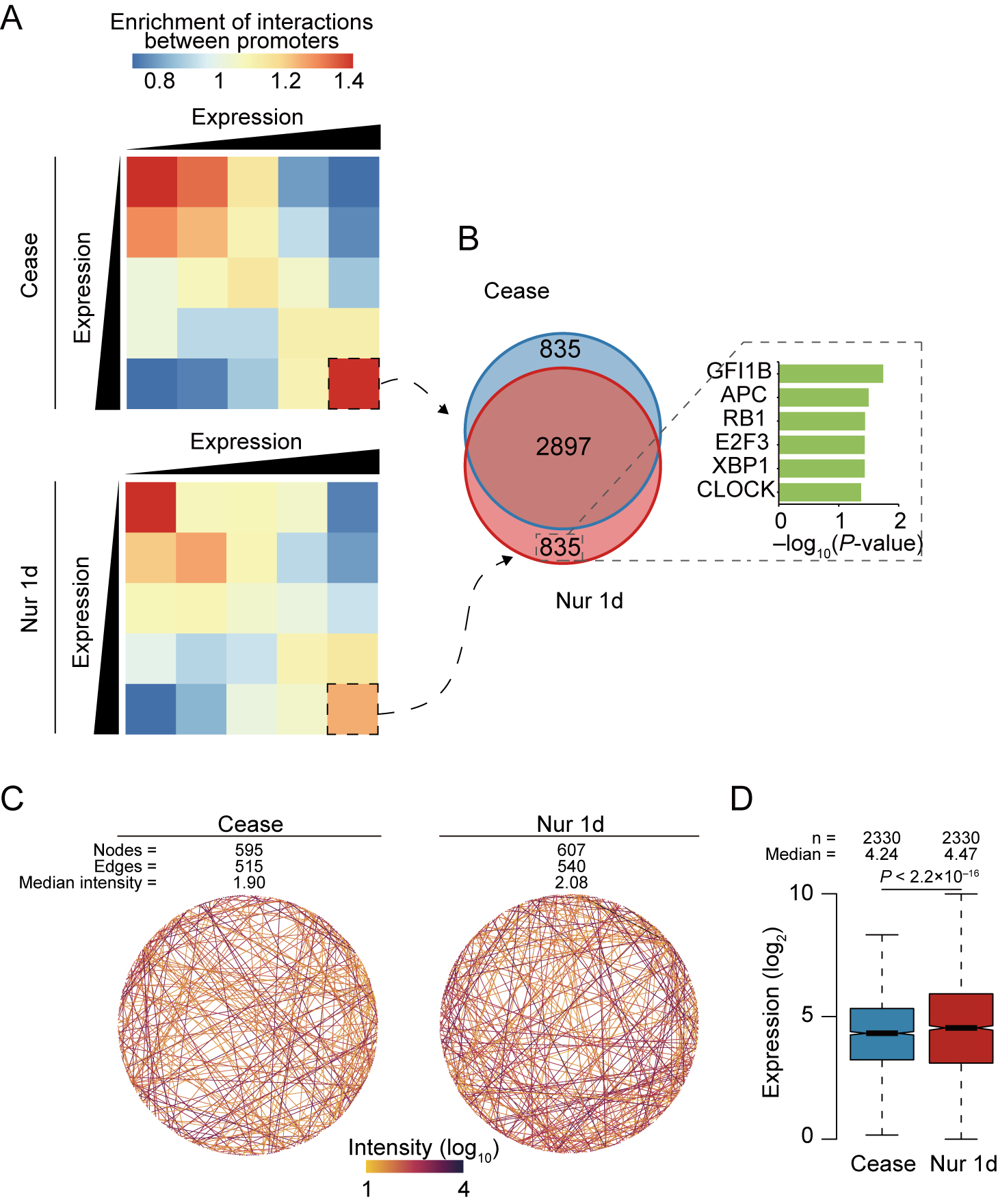


**S4-V Appendix. Promoter-promoter interaction network between Ceased stage and Nur 1d. A.** Enrichment of interactions between promoters from different expression categories. **B.** Venn diagram showing the distribution of top 20% genes with the highest expression levels in Cease and Nur1d (left panel), and enrichment of transcription factors (TFs) specific to Nur1d genes using Enrichr software (http://amp.pharm.mssm.edu/Enrichr). **C.** Promoter-promoter interaction network of STAT5 target genes based on intra-chromosomal matrix. The color of the edge represents interaction intensity. **D.** Expression levels of STAT5 target genes. Human STAT5 target genes are obtained from hTFtarget [2] database, and converted to chicken orthologs, and pigeon orthologs.

**Reference**

1. Krijger PH, Di Stefano B, de Wit E, Limone F, van Oevelen C, de Laat W, et al. Cell-of-origin-specific 3D genome structure acquired during somatic cell reprogramming. Cell Stem Cell. 2016;18(5):597-610.

2. Zhang Q, Liu W, Zhang HM, Xie GY, Miao YR, Xia M, et al. hTFtarget: A comprehensive database for regulations of human transcription factors and their targets. Genom Proteom Bioinf. 2020;18(2):120-128.
